# CCT3-*LINC00326* axis regulates hepatocarcinogenic lipid metabolism

**DOI:** 10.1136/gutjnl-2021-325109

**Published:** 2022-01-12

**Authors:** Jonas Nørskov Søndergaard, Christian Sommerauer, Ionut Atanasoai, Laura C Hinte, Keyi Geng, Giulia Guiducci, Lars Bräutigam, Myriam Aouadi, Lovorka Stojic, Isabel Barragan, Claudia Kutter

**Affiliations:** 1 Department of Microbiology, Tumor, and Cell Biology, Science for Life Laboratory, Karolinska Institute, Stockholm, Sweden; 2 Barts Cancer Institute, Centre for Cancer Cell and Molecular Biology, John Vane Science Centre, Queen Mary University of London, London, UK; 3 Comparative Medicine, Karolinska Institute, Stockholm, Sweden; 4 Department of Medicine, Karolinska Institute, Stockholm, Sweden; 5 Department of Physiology and Pharmacology, Karolinska Institute, Stockholm, Sweden

**Keywords:** lipid metabolism, hepatocellular carcinoma

## Abstract

**Objective:**

To better comprehend transcriptional phenotypes of cancer cells, we globally characterised RNA-binding proteins (RBPs) to identify altered RNAs, including long non-coding RNAs (lncRNAs).

**Design:**

To unravel RBP-lncRNA interactions in cancer, we curated a list of ~2300 highly expressed RBPs in human cells, tested effects of RBPs and lncRNAs on patient survival in multiple cohorts, altered expression levels, integrated various sequencing, molecular and cell-based data.

**Results:**

High expression of RBPs negatively affected patient survival in 21 cancer types, especially hepatocellular carcinoma (HCC). After knockdown of the top 10 upregulated RBPs and subsequent transcriptome analysis, we identified 88 differentially expressed lncRNAs, including 34 novel transcripts. CRISPRa-mediated overexpression of four lncRNAs had major effects on the HCC cell phenotype and transcriptome. Further investigation of four RBP-lncRNA pairs revealed involvement in distinct regulatory processes. The most noticeable RBP-lncRNA connection affected lipid metabolism, whereby the non-canonical RBP CCT3 regulated *LINC00326* in a chaperonin-independent manner. Perturbation of the CCT3-*LINC00326* regulatory network led to decreased lipid accumulation and increased lipid degradation *in cellulo* as well as diminished tumour growth *in vivo*.

**Conclusions:**

We revealed that RBP gene expression is perturbed in HCC and identified that RBPs exerted additional functions beyond their tasks under normal physiological conditions, which can be stimulated or intensified via lncRNAs and affected tumour growth.

**Significance of this study**

**What is already known on this subject?**

- RNA-binding proteins (RBPs) play crucial roles in cancer.
- RBPs can function through long non-coding RNAs (lncRNAs).
- Cell type-specific expression patterns of lncRNAs have strong diagnostic, prognostic and therapeutic value.

**What are the new findings?**

- Integrative multiomics approach, including a new liver cancer cohort dataset, describes the transcriptional landscape in hepatocellular carcinoma (HCC).
- RBPs are significantly deregulated in HCC mounting in reduced patient survival.
- Pathological RBPs control metabolic activity and apoptosis in HCC cells through lncRNAs.
- Chaperonin complex subunit CCT3 moonlights as a novel RBP.
- CCT3 functions in HCC lipid metabolism via the long non-coding RNA *LINC00326 in cellulo* and *in vivo*.

**How might it impact on clinical practice in the foreseeable future?**

- Diagnostic and prognostic potential of HCC stage-specific response of CCT3 and *LINC00326.*
- Beneficial therapeutic effects of lncRNA overexpression at a specific HCC disease stage.

## Introduction

Liver cancer encompasses a collection of clinically diverse tumour subtypes that arise from malignant liver cells. Hepatocellular carcinoma (HCC) is the most common form affecting ~80% of all patients.^1^ The cause of HCC is often attributed to intrinsic, extrinsic and unknown idiopathic factors.^2^ Perturbation of cellular homeostasis leading to uncontrolled cell growth and proliferation is characteristic for HCC but the underlying molecular consequences are only partly understood. In recent years, more focus has been placed on studying deregulated long non-coding RNAs (lncRNAs) in cancer.^3 4^ However, functionality of many lncRNAs still remains to be explored. LncRNAs are transcripts longer than 200 nucleotides that either originate from intergenic regions (lincRNAs) or coincide within transcriptional units of different genes.^5^ Given the unique cell type- and disease-specific expression patterns of lncRNAs,^6 7^ they are emerging targets for biomarker and therapeutic developments since their presence affects primarily the diseased cell. LncRNAs regulate various processes, such as cell cycle, proliferation, apoptosis and cell death.^5^ These actions are likely mediated through interaction with RNA-binding proteins (RBPs).^8^

As vital enzymes, RBPs control RNA regulatory pathways.^9^ RBP activity is adjusted to the cellular demand of RNA transcripts. Altering RBP gene expression levels has profound implications on cellular physiology and contributes to the phenotypic abnormalities commonly observed in atypical and cancer cells.^10^ RBPs contain known or predicted RNA-binding domains (RBD). For example, the canonical RBP Insulin Like Growth Factor 2 mRNA Binding Protein 1 (IGF2BP1) contains six RBDs through which IGF2BP1 regulates mRNA stability, such as, by impeding access of miRNAs to their targets.^11^ Over 500 proteins with classical RBDs have been identified in human cells.^12^ Moreover, additional proteins with RNA-binding capacity were found through newer technologies (online supplemental table S1). While these non-canonical RBPs have well-established biological functions, they can also moonlight as RBPs.^13^ For instance, Tripartite Motif 25 (TRIM25) ubiquitinates proteins for degradation^14^ and as a non-canonical RBP binds RNA to regulate innate immune response pathways.^14^ Likewise, Alpha Enolase (ENO1) is indispensable in glycolysis, and its enzymatic activity is abolished through the interaction with a lncRNA.^15^

10.1136/gutjnl-2021-325109.supp1 Supplementary data

Due to the frequent dependency between lncRNAs and RBPs,^8^ we here used a novel RBP-centric approach to identify functional lncRNAs. We curated a list of 2282 RBPs reported in RNA interactome capture experiments across different cell types (online supplemental table S1) and found aberrant RBP gene expression profiles in two HCC patient cohorts. Perturbation of selected RBPs in *in cellulo* and *in vivo* settings revealed the underlying regulatory networks through which RBPs can act. Our approach led to the identification of new functional lncRNAs and revealed their regulatory roles in HCC. Specifically, we found that *LINC00326* regulates lipid metabolism through its interaction with the non-canonical RBP CCT3.

## Results

### Differentially expressed RNA-binding proteins impact survival of patients with hepatocellular carcinoma

**Figure 1:**
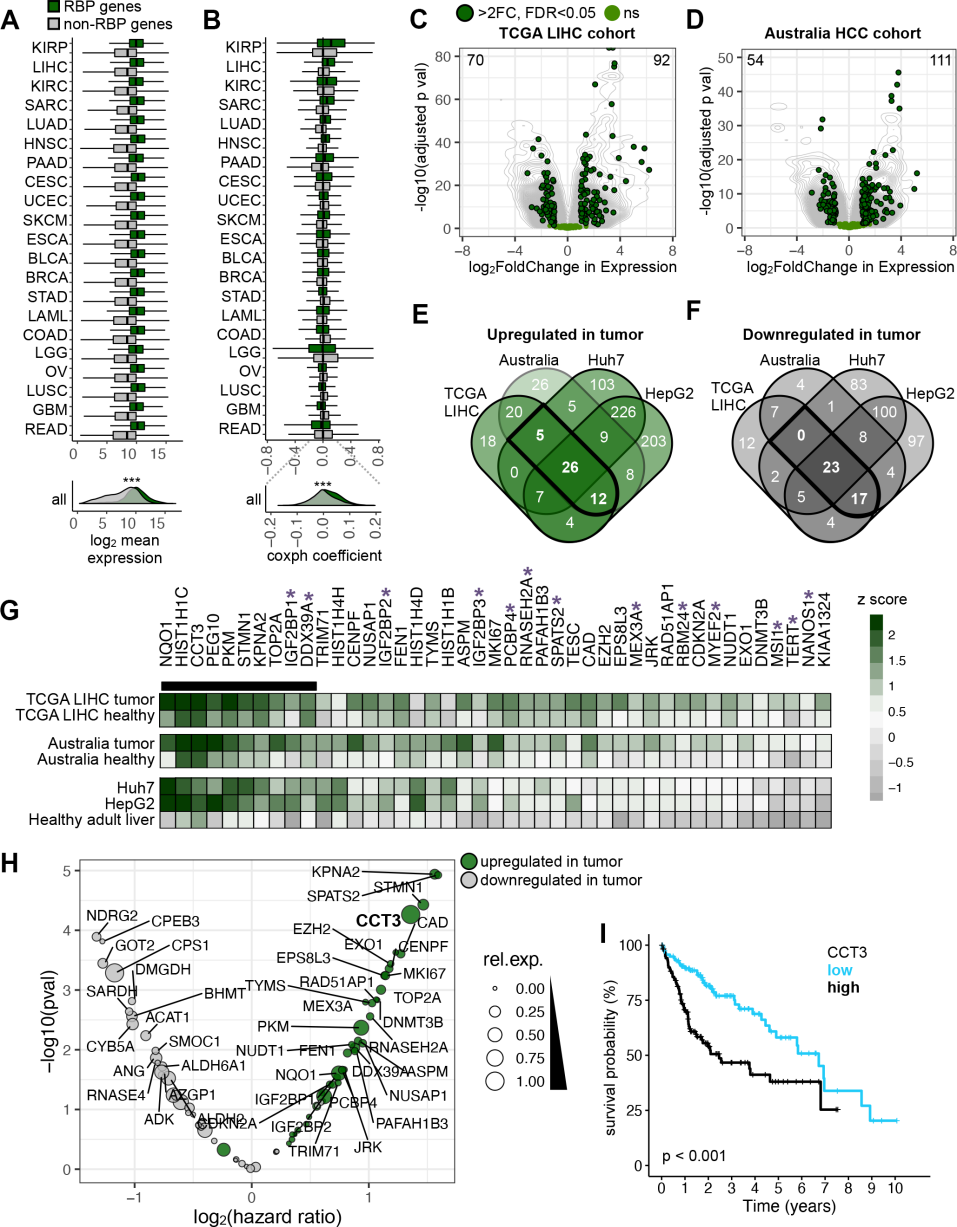
RBPs are deregulated in cancer and affect patient survival. (A, B) Boxplots of (A) gene expression level and (B) cox proportional hazard (coxph) coefficient of protein-coding genes grouped into RBP (green) and non-RBP (grey) genes. Each row represents a different cancer type defined by TCGA. Each cancer type consisted of 144 to1006 patients. Hinges correspond to the first and third quartiles, and whiskers correspond to the 1.5-times interquartile range. (C, D) Volcano plots demonstrate differentially expressed (DE) genes in the two HCC cohorts (C) TCGA-LIHC and (D) Australia HCC. Data points represent DE RBP genes (dark green), not significantly (ns)-DE RBP genes (FDR>0.05, light green) and all other genes (grey). (E, F) Four-way Venn diagrams intersect the number of (E) upregulated and (F) downregulated RBP genes in the TCGA and Australia HCC cohorts as well as in liver cancer cell lines Huh7 and HepG2. Intersections highlighted (bold) show the number of RBP genes commonly deregulated in HCC cohorts and cell lines. (G) Heatmap displays changes in expression levels for commonly upregulated RBP genes in HCC as highlighted in (E). Expression level is sorted by average tumour z-score from left to right. Black bar marks the top 10 highest expressed RBP genes, and purple asterisk marks RBPs with a canonical RBD. Colour gradient indicates z-score differences (green: high; grey: low). (H) Volcano plot displays comparison of the top and bottom tercile in RBP gene expression levels and hazard ratioswithin the TCGA-LIHC cohort (377 patients) (grey: downregulated and green: upregulated in tumour). The size of the circle represents the gene expression level of each RBP relative to each other (broad: high, narrow: low). (I) Kaplan-Meier plot shows the association of *CCT3* gene expression level and 10-year survival within the top and bottom tercile within the TCGA-LIHC cohort (377 patients) (black: high and blue: low *CCT3* gene expression levels). Statistics: log-rank (Mantel-Cox). BLCA, bladder urothelial carcinoma; BRCA, breast invasive carcinoma; CESC, cervical squamous cell carcinoma and endocervical adenocarcinoma; COAD, colon adenocarcinoma; DE, differentially expressed; ESCA, oesophageal carcinoma; GBM, glioblastoma multiforme; HCC, hepatocellular carcinoma; HNSC, head and neck squamous cell carcinoma; KIRC, kidney renal clear cell carcinoma; KIRP, kidney renal papillary cell carcinoma; LAML, acute myeloid leukaemia; LGG, brain lower grade glioma; LIHC, liver hepatocellular carcinoma; LUAD, lung adenocarcinoma; LUSC, lung squamous cell carcinoma; OV, ovarian serous cystadenocarcinoma; PAAD, pancreatic adenocarcinoma; RBP, RNA-binding proteins; READ, rectum adenocarcinoma; SARC, sarcoma; SKCM, skin cutaneous melanoma; TCGA, The Cancer Genome Atlas; UCEC, uterine corpus endometrial carcinoma.

Deregulation of genes is a key feature of cancer. Due to their regulatory capacity, genes encoding for RBPs have gained more attention.^10^ We therefore built a comprehensive catalogue of genes with RNA-binding capacity in human by inspecting RNA interactome capture experiments and gene ontology (GO) databases (online supplemental table S1). The resulting list comprised 1321 canonical (containing known RBDs) and 959 non-canonical (without any characterised RBDs) RBPs (online supplemental table S1). We compared gene expression levels and survival probabilities of RBP *versus* all other protein-coding genes across 21 different human cancer types (The Cancer Genome Atlas, TCGA).^16^ RBP gene expression was higher irrespective of the cancer type (two-tailed unpaired student’s t-test, p<0.001) (figure 1A), which is in accordance to previous estimates.^17^ Division of RBPs into canonical and non-canonical RBPs yielded the same results (online supplemental figure S1a). To investigate the impact of RBP deregulation on patient survival, we compared the cox proportional hazard coefficient (coxph) of RBP and non-RBP genes for each cancer type.^16^ We found the largest fold change (FC) in HCC (LIHC, 6.3 FC) followed by sarcoma (SARC, 5.1 FC) (figure 1B). Kidney cancers (KIRP and KIRC) had relatively high absolute coxph values for RBP and non-RBP genes and thus lower FCs (4.2 and 1.6, respectively) than the other top-ranked cancer types. Therefore, RBP gene expression had a greater prognostic value in liver cancer than any other investigated cancer type, which might be explained due to high proliferation rate^17^ and global activation of RBP gene copies.^18^ By comparing paired data of tumour and peritumour tissue from 50 LIHC cohort patients (online supplemental figure S1b), we found 92 upregulated and 68 downregulated RBP genes (figure 1C, online supplemental table S2).

To validate our findings in a TCGA-independent HCC cohort, we profiled matched pairs of primary tumour and peritumour liver samples of 24 patients with HCC from the Australian Victorian Cancer Biobank (Australia HCC) by RNA-sequencing (RNA-seq) (figure 1D). Similar to the TCGA LIHC cohort, the expression pattern separated the tumour and peritumour tissue samples in the Australia HCC cohort (online supplemental figure S1c). Our differential gene expression analysis revealed 111 upregulated and 64 downregulated RBP genes (figure 1D–F, online supplemental tables S3 and S4). In both cohorts, we identified a common set of 63 upregulated and 47 downregulated RBP genes (figure 1E, F). We complemented our analysis by including gene expression data of the human HCC cell lines HepG2 and Huh7.^19^ A total number of 26 upregulated and 23 downregulated RBP genes showed similar gene expression patterns between all HCC datasets (figure 1E, F, online supplemental figure S1e).

We ranked z-score gene expression values across all datasets and selected the top 10 highest expressed canonical and non-canonical RBPs that were upregulated in HCC (*NQO1*, *HIST1H1C*, *CCT3*, *PEG10*, *PKM*, *STMN1*, *KPNA2*, *TOP2A*, *IGF2BP1*, *DDX39A*) (figure 1G). By analysing Cap Analysis of Gene Expression transcriptome data,^20^ we confirmed upregulation of these RBP genes in HCC (online supplemental figure S1d). The selected RBPs have paralogous genes and belong to different protein families that are composed of diverse predicted protein domains (online supplemental figure S1f). Protein expression of these RBPs was detectable in multiple subcellular compartments (online supplemental figure S1g).

These upregulated RBP genes carried a spectrum of mutations and copy number variations in HCC. In particular, Chaperonin Containing TCP1 Subunit 3 (*CCT3*) was frequently amplified (online supplemental figure S1h). We inspected the hazard ratio (HR) and found that high RBP gene expression levels were associated with poor patient survival (figure 1H). The majority (7/10) of RBPs had a significant prognostic value (corrected for tumour stage, age and gender) (figure 1H, I and online supplemental figure S2). In comparison to all RBPs, high expression of Karyopherin Subunit Alpha 2 (*KPNA2*) had the largest HR closely followed by *CCT3* and Stathmin 1 (*STMN1*) (figure 1H). Within their respective gene families, *CCT3*, *IGF2BP1*, *KPNA2*, NAD(P)H Quinone Dehydrogenase 1 (*NQO1*), and *STMN1* had the highest HR, while DExD-Box Helicase 39A (*DDX39A*), Histone Cluster 1 H1 Family Member C (*HIST1H1C*), Paternally Expressed 10 (*PEG10*) and DNA Topoisomerase II Alpha (*TOP2A*) had gene family members with a higher HR (online supplemental figure S3a). Elevated gene expression levels for most family members was associated withdecreased patient survival (figure 1I, online supplemental figure S3b, c). Similar results were previously obtained when dividing *CCT3* gene expression levels into halves^21^ instead of terciles.

In summary, we identified a set of RBPs belonging to diverse gene families that were deregulated in HCC. RBP gene expression levels could therefore be used as potential prognostic markers in HCC patients.

### RBP knockdown reduces cancer growth and changes non-coding RNA expression in HCC cell lines

**Figure 2:**
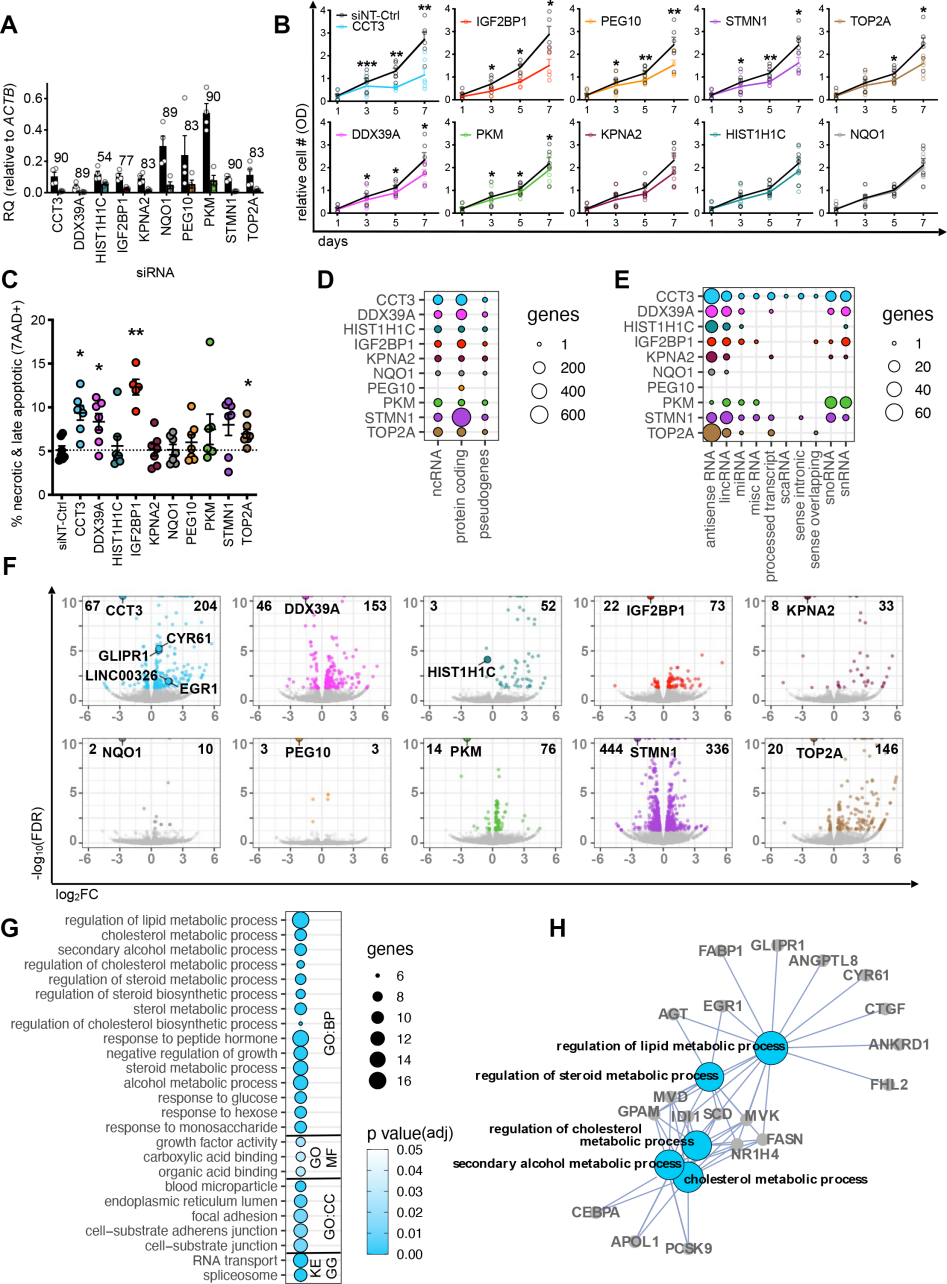
Reduced expression of RBP genes impact various cellular and molecular responses in HCC cell lines. (A) Bar graph displays RBP gene expression levels before (black bars) and after siRNA-mediated RBP-KD (coloured bar) relative to *ACTB* in HepG2 and Huh7 cells determined by qPCR (n=4 mean, ±SEM). Number above each bar shows the average KD efficiency in percent. Colour code: *CCT3* (blue), *DDX39A* (magenta), *HIST1H1C* (turquoise), *IGF2BP1* (red), *KPNA2* (plum), *NQO1* (grey), *PEG10* (yellow), *PKM* (green), *STMN1* (purple) and *TOP2A* (brown). Individual replicates are displayed by white circles. (B) Line graphs show the relative number of metabolically active cells (measured by optical density) over 7 days after siRNA-mediated RBP-KDs assayed by the MTT assay (n=5). Black line: non-targeting siRNA control (siNT-Ctrl), coloured line: RBP-specific siRNA KD. (C) Dot plot represents the percentage of dead cells after siRNA-mediated RBP-KDs (colour-coded) after 5 days (n=7, mean, ±SEM). (B, C) Statistics: paired two-tailed t-test, *p<0.05, **p<0.01, ***p<0.001. (D, E) Circle plots display number of genes per RNA biotype affected by RBP-KDs. The diameter of the circles corresponds to the number of genes in each category. Deregulated genes falling into different ncRNA subcategories are shown in figure 2E.(F) Volcano plots demonstrate DE genes after RBP-KDs. Data points represent significantly DE genes (colour-coded by RBP-KD, FDR<0.05) and not significantly DE genes (grey, FDR>0.05). Bolded numbers on the top of each graph indicate total number of DE genes (FDR<0.05). The wider circle in each plot highlights the downregulated RBP. Genes with an FDR value smaller than 1 × 10^−10^ were collapsed at 1 × 10^−10^. (G) Circle plot shows GO term and KEGG pathway enrichment analysis of deregulated genes after the *CCT3*-KD (FDR<0.05). The diameter of the circles corresponds to the number of genes in each GO or KEGG term and the colour code represents varying degrees of significance (white: high and blue: low p value). (H) Interaction network displays connections of the five most significant GO BP terms in figure 2G. GO term is bolded and gene names are highlighted. BP, biological process; CC, cellular compartment; DE, differentially expressed; KD, knockdown; MF, molecular function; OD, optical density; RQ, relative quantity.

In order to dissect the functional roles of upregulated RBP genes in the HCC cohorts and cell lines, we performed siRNA-mediated gene knockdown (KD) in Huh7 and HepG2 (figure 2, online supplemental figure S4a–f). We achieved high RBP-KD efficiencies and confirmed a mean reduction of 80% (range: 54%–90%) by RT-qPCR (figure 2A). For the majority (7/10) of RBPs, the KDs resulted in a significant decrease in the number of metabolically active cells 3 days post-transfection (MTT assay) (figure 2B) due to an increase of cell death (FACS for late apoptosis/necrosis) (figure 2B, C), especially after KD of *CCT3* and *IGF2BP1*.

To investigate the molecular mechanisms underlying the observed cellular phenotypes of the RBP-KDs, we profiled gene expression by RNA-seq. Each RBP-KD affected different annotated RNA biotypes (12 different categories) (figure 2D–G, online supplemental tables S5–S7). The majority of differentially expressed (DE) genes were protein-coding (mean: 120, range: 6–697) followed by non-coding (mean: 44, range: 0–105) and pseudogenes (mean: 6, range: 0–18) (figure 2D). Closer inspection of the non-coding RNA biotype revealed that genes encoding for antisense and lincRNAs were frequently deregulated after the RBP-KD (figure 2E). The *CCT3*-KD showed the highest diversity in RNA biotypes and affected the highest number of non-coding genes (n=105) among all RBPs tested. The *STMN1*-KD led to the highest (n=780), while the *PEG10*-KD led to the lowest (n=6) number of DE genes (figure 2F). The number of DE genes observed in our RBP-KD experiments is in accordance to previous shRBP-KD experiments (n=235) in K562 cells (online supplemental figure S4g).^22 23^ To understand whether the altered expression of RBP genes impacts specific regulatory processes, we performed a GO term and KEGG pathway analysis of the DE genes for each RBP-KD (figure 2G, H, online supplemental figure S5, online supplemental table S8). Deregulated genes belonged to GO terms distinct for each RBP-KD, e.g., lipid metabolism (*CCT3*-KD), angiogenesis (*DDX39A*-KD), response to oxygen levels (*IGF2BP1*-KD), RNA transport (*PKM*-KD), lipid localisation and transport (*STMN1*-KD) and DNA packaging and conformation change (*TOP2A*-KD) (online supplemental figure S5, online supplemental table S8).

In sum, deregulation of some RBPs in HCC caused diverse alteration of gene regulatory programmes linked to metabolism and lipogenesis.

### Each RBP-KD is accompanied by gene expression changes of a specific set of annotated and novel lincRNA genes

**Figure 3:**
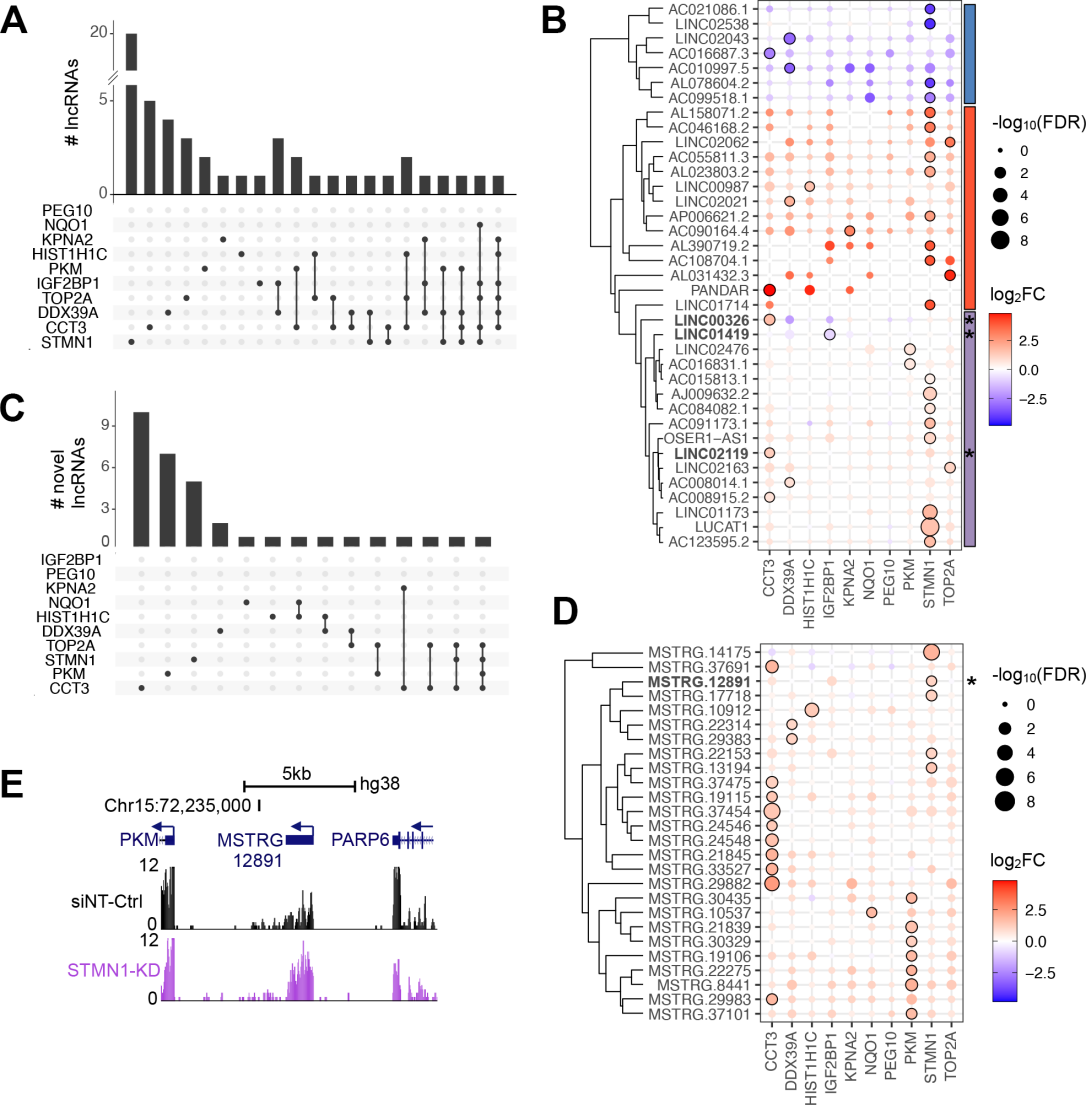
RBP-KD affects lincRNA gene expression levels. (A, C) Bar graph show the number of (A) annotated and (C) novel DE lincRNA genes detectable after RBP-KDs. The frequency of lincRNA genes in one (black dot) or multiple (black dots connected by a line) RBP-KD experiments is shown. (B, D) Circle plots display the occurrence of (B) annotated and (D) novel lincRNA genes per RBP-KD. The diameter of the circles corresponds to varying degrees of significance (large: high, and narrow: low FDR value, black line: FDR<0.05). The colour code represents fold change (red: upregulated and blue: downregulated). Vertical bars specify the three most common clusters defining lincRNAs as either consistently downregulated (blue) or upregulated (red), or with varying pattern deregulation across the ten RBP-KD (purple). A star (*) marks lincRNAs used for further investigation. (E) The UCSC genome browser view demonstrates the genomic location of the novel lincRNA *MSTRG.12891* in between genes encoding for *PKM* and *PARP6*. Arrows indicate direction of gene transcription. Gene expression patterns in Huh7 cells transfected with siNT-Ctrl (black) or siRNA-mediated KD of *STMN1* (purple) are shown. The y-axis of each track specifies normalised RNA-seq read intensity.

Since GO terms are largely curated based on information obtained from protein-coding genes, the functional association of deregulated non-coding RNAs remains uncertain. Beyond protein-coding genes, we found 54 deregulated annotated lincRNAs (figure 3A) of which 69% (37/54) and 31% (17/54) were deregulated in one or more RBP-KDs, respectively. LincRNAs were more up- (76%, 41/54) than downregulated (24%, 13/54) after RBP-KDs (figure 3B). Among the upregulated lincRNAs were Lung Cancer Associated Transcript 1 (*LUCAT1*) and Promoter of CDKN1A Antisense DNA Damage Activated RNA (*PANDAR*), which have been linked to various cancer types.^24 25^ Hierarchical clustering of the annotated deregulated lincRNAs revealed three distinct groups of commonly up- and downregulated lincRNAs as well as lincRNAs that were specifically affected in a single RBP-KD experiment (figure 3B). We expected that lincRNAs in the latter group have a specific rather than a general role in cancer pathways (figure 3B).

At the point of the analysis, 7307 lincRNA genes had been annotated in the human genome (Gencode GRCh38 v.27). To identify novel lincRNAs that were only detectable after RBP-KDs, we *de novo* annotated lincRNA in our RNA-seq data and found 34 novel lincRNAs that were upregulated in at least one RBP-KD (figure 3C–E, online supplemental table S9). It is possible that those novel lincRNAs have been undetected previously due to rapid turnover in the presence of highly abundant RBPs.

Overall, our data-driven approach revealed that the majority of novel lincRNAs were specific for one RBP-KD, in particular after the *CCT3*-KD (figure 3D).

### Overexpression of lincRNA genes alters cancer cell phenotypes towards early apoptosis

**Figure 4:**
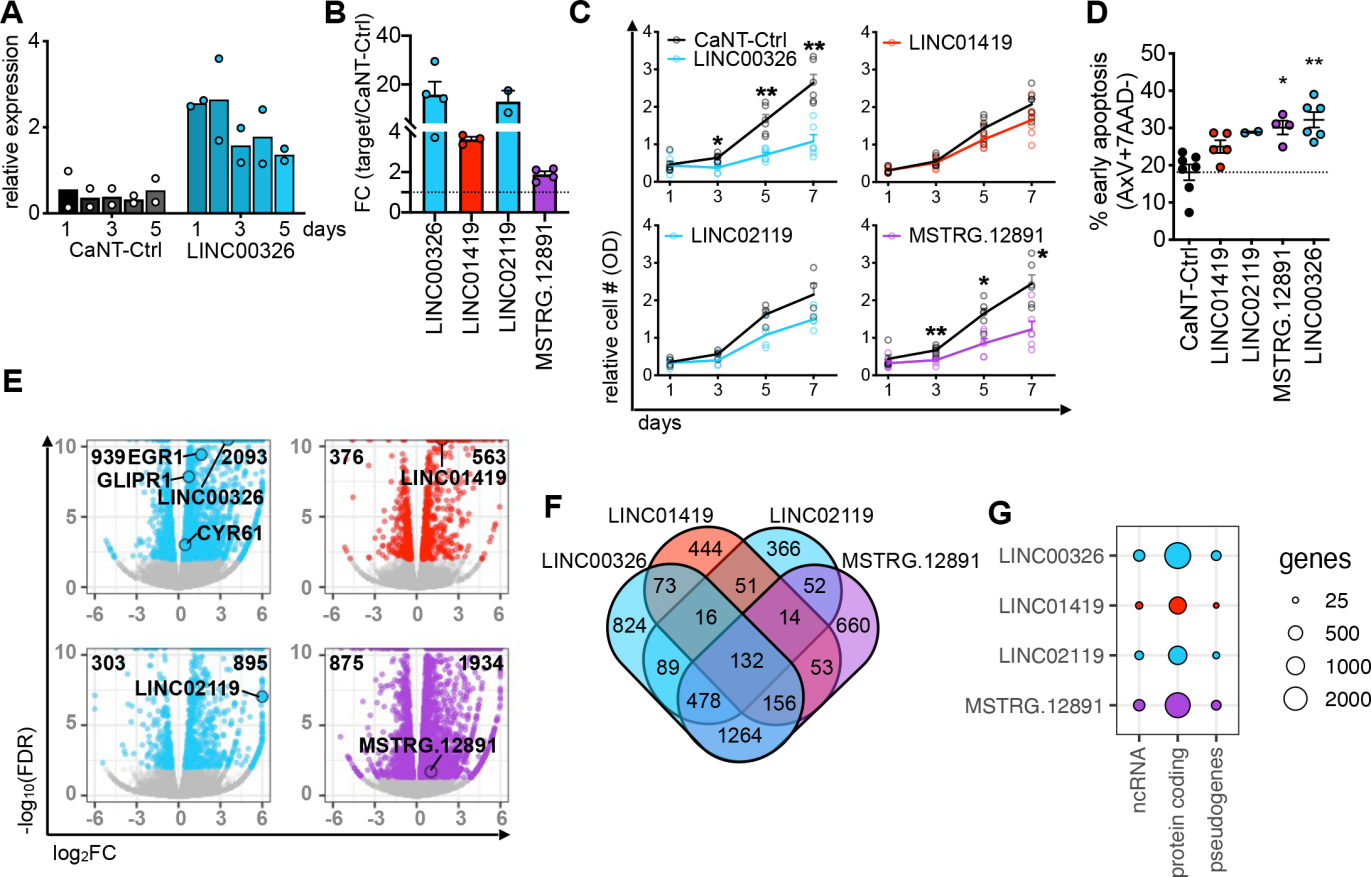
Overexpression of lincRNA genes in HCC cell line causes molecular and cellular alterations. (A) Bar graphs exemplify the increase in relative gene expression of *LINC00326* over 5 days after transfection of a CRISPRa vector with non-targeting gRNA-CRISPRa controls (CaNT-Ctrl, black gradient) or three lincRNA-specific gRNAs (*LINC00326*, blue gradient) determined by RT-qPCR. (B) Bar graph demonstrate the fold change in lincRNA gene expression 2 days after CRISPRa transfection of lincRNA-specific *versus* CaNT-Ctrl determined by RT-qPCR (n=2–4, mean, +SEM). The colour-code links the lincRNA to the respective RBP-KD experiment in which the lincRNA was identified (blue: *CCT3*, red: *IGF2BP1*, purple: *STMN1*). (C) Line graphs show relative increase in metabolically active HCC cell number over 7 days after CRISPRa transfection with CaNT-Ctrl (black) or lincRNA-specific gRNAs (coloured) determined by MTT assay. (D) Dot graph shows the percentage of early apoptotic cells 5 days after CRISPRa transfection with CaNT-Ctrl (black) and lincRNA-specific gRNAs (coloured) determined by FACS (n=2–8, mean, ±SEM). (A–D) Each biological replicate of Huh7 and HepG2 is displayed by circles. Graphs are coloured according to the colour-code selected for the RBP partner through which the lincRNA was identified (blue: *CCT3*, red: *IGF2BP1*, purple: *STMN1*). Statistics: paired two-tailed t-test, *p<0.05, **p<0.01. (E) Volcano plots demonstrate DE genes 2 days after CRISPRa transfection of lincRNA-specific *versus* CaNT-Ctrl determined by RNA-seq in Huh7. Data points represent significantly DE genes (coloured, FDR<0.01) and not significantly DE genes (grey, FDR>0.01). Bolded numbers on the top of each graph indicate total number of DE genes (FDR<0.01). Circle highlights lincRNA and lincRNA-interacting genes investigated. (F) Four-way Venn diagram intersects the number of DE genes after each lincRNA-OE experiment (FDR<0.01). (G) Circle plot displays the number of genes per RNA biotype affected by the lincRNA-OE. The diameter of the circles corresponds to the number of genes in each category.

To further characterise the roles of lincRNAs in HCC, we applied stringent selection criteria including (1) FC in lincRNA gene expression, (2) RBP-specific dependency, (3) lincRNA abundance and (4) visual inspection in the genome browser. This led to a subsequent analysis of four lincRNAs, *LINC00326*, *LINC01419, LINC02119* and *MSTRG.12891*. Since lower RBP gene expression levels resulted in increased expression levels of lincRNA genes (figure 3B, D), we hypothesised that an increase in lincRNA gene expression reveals a liver cancer cell-specific phenotype. We used CRISPR-VPR activation (CRISPRa) with three target-specific guide RNAs (gRNAs) to overexpress selected lincRNA genes (lincRNA-OE) and achieved a 2-fold to 20-fold increase of lincRNA gene expression that lasted for several days (figure 4A, B).

In all four lincRNA-OE experiments, we detected a reduced number of metabolically active Huh7 and HepG2 cells and early cell apoptosis, which was significant for *LINC00326* and *MSTRG.12891* (figure 4C, D, online supplemental figure S6a–e). To explain the phenotype, we performed RNA-seq. Overall, lincRNA-OEs resulted in more upregulation than downregulation of genes (figure 4E, online supplemental figure S6f, online supplemental table S10–12) and many of the deregulated genes were specific for the respective lincRNA-OE (figure 4F). In contrast to the RBP-KDs, lincRNA-OEs resulted in frequent alteration of protein-coding genes and only few changes in ncRNA and pseudogene expression (figure 4G, online supplemental figure S6g–h). We performed GO term and KEGG pathway analyses (online supplemental figure S7, online supplemental table S13) and found an enrichment in lipid transporter activity after *LINC00326*-OE and *MSTRG.12891*-OE and growth factor binding after *LINC01419*-OE and *LINC02119*-OE.

In conclusion, lincRNA-OEs resulted in increased early apoptosis and reduced metabolic activity as well as transcriptional alteration of specific biological pathways. In particular, we found the strongest perturbation when increasing gene expression of CCT3-dependent *LINC00326* in lipid metabolism.

### A CCT3-*LINC00326* network regulates lipid metabolism

**Figure 5:**
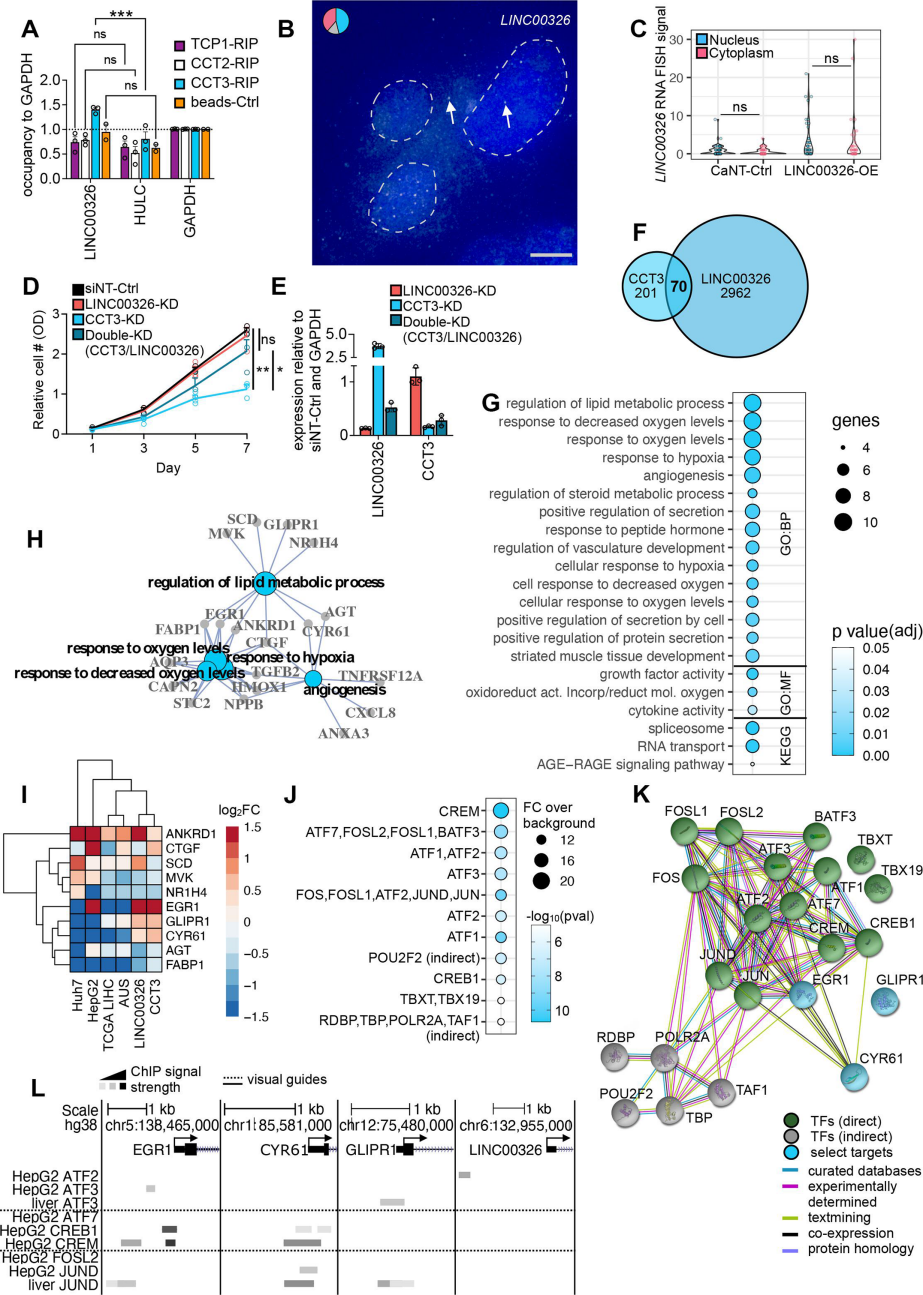
The CCT3-*LINC00326* interactome regulates lipid metabolism. (A) Bar graph shows enrichment of *LINC00326* compared with GAPDH and HULC (negative controls) over input control after RNA immunoprecipitation with a TCP1 (CCT1)-(purple), CCT2- (white) or CCT3-specific (blue) antibody *versus* the beads-only (no antibody) control (orange) followed by RT-qPCR with gene- and strand-specific primers (n=3, mean, +SEM). Each biological replicate is displayed by circles. Statistics: ANOVA with a Bonferroni’s multiple comparison test, ***p<0.001, ns: non-significant. (B) Microscopic image of single-molecule RNA FISH using exonic probes for *LINC00326* (white dots and arrows) in *LINC00326*-OE Huh7 HCC cells. DAPI (blue) marks the nucleus. Pie chart represents the fraction of signals in the nucleus (blue) or cytoplasm (pink) in cells, or cells without any signal (grey). Scale bar: 5 µm. (C) Violin plots show quantification of *LINC00326* RNA FISH signal localisation in Huh7 HCC cells (n=40–53, statistics: paired two-tailed t-test, ns: non-significant). (D) Line graphs shows the relative number of metabolically active cells (measured by optical density, OD) over 7 days after the siRNA-mediated *CCT3*-KD and/or *LINC00326*-KD assayed by MTT assay (n=3, mean, +SEM, statistics: ANOVA with a Bonferroni’s multiple comparison test, *p<0.05, **p<0.01). (E) Bar charts show siRNA-KD-efficiencies of targeted RBP genes (figure 5D) (n=3, mean, +SEM). (F) Two-way Venn diagram intersects the number of deregulated genes after *CCT3*-KD and *LINC00326*-OE. (G) Circle plot shows GO term and KEGG pathway enrichment analysis of the 70 commonly deregulated genes after *CCT3*-KD and *LINC00326*-OE. The diameter of the circles corresponds to the number of genes in each GO or KEGG term and the colour code represents varying degrees of significance (white: high and blue: low p value). (H) Interaction network displays connections of the five most significant GO BP terms shown in figure 5G. GO term is bolded and gene names are highlighted. (I) Heatmap (unsupervised clustering) displays the fold change in expression levels for lipid metabolic process genes (figure 5H) when comparing HCC cohorts and cell lines, *CCT3*-KD and *LINC00326*-OE over non-cancerous or NT controls, respectively. Colour gradient indicates log_2_FC differences (red: high; blue: low). (J) Circle plot demonstrates enrichment of TF-binding motifs in the promoter regions of the 70 commonly deregulated genes of the *CCT3*-KD and *LINC00326*-OE. The diameter of the circles corresponds to the fold change over background controls and the colour code represents varying degrees of significance (white: high and blue: low p value). Identified motifs for each TF are shown in online supplemental figure S9f. (K) StringDB interaction network shows the links of the TFs identified in figure 5F and known interaction partners (direct: green, indirect: grey). Two direct connections to lipid metabolism genes are highlighted (blue). (L) The UCSC genome browser view demonstrates genomic location of three lipid metabolism-associated genes and the *LINC00326* gene. Arrows indicate direction of gene transcription. Horizontal bars indicate ChIP-seq signals (black: strong; grey: weak) for available TF-binding events in HepG2 or liver cells (ENCODE).

Due to the strongest phenotypical severity, we further investigated the interaction of CCT3 and *LINC00326*. Afteroverexpression, we found that *LINC00326* but not the liver-specific control lincRNA *HULC* was coimmunoprecipitated with CCT3 in liver cancer cells (figure 5A). Other components of the chaperonin-complex did not coimmunoprecipitate with *LINC00326*, thus indicating a chaperonin-independent function. Likewise, KD of these chaperonin-components did not lead to *LINC00326* upregulation (online supplemental figure S8a, b). In addition, CCT3 protein and *LINC00326* were located in the same cellular compartment enabling their interaction (figure 5B, C, online supplemental figures S1g, S8c–e,^26^). *CCT3* RNA stoichiometry data further suggested an enrichment of a chaperonin-independent function in HCC when compared with healthy tissue and 14 other cancer types (online supplemental figure S8f). Remarkably, the double-KD of *CCT3* and *LINC00326* rescued the *CCT3*-KD phenotype, further verifying their functional dependence (figure 5D, E). Functional inspection of the 70 commonly deregulated genes of the *CCT3*-KD and *LINC00326*-OE (figure 5F) suggested an involvement in the regulation of lipid metabolic processes, response to decreased oxygen levels and angiogenesis (figure 5G–H). Lipids are degraded via peroxidation.^27^ Accordingly, a balanced cellular oxygen supply is interrelated with processes regulating vascularisation, such as angiogenesis.^28^ None of the other RBP-lincRNA interactions assayed in this study acted through these biological pathways (online supplemental figure S9a–e). Most genes (8/10) were regulated in similar directions in the *CCT3*-KD and the *LINC00326*-OE (figure 5I). When including our HCC cohort and HCC cell line datasets (figure 1E, F), we noticed that a cluster consisting of genes encoding for Early growth response protein 1 (*EGR1*), Glioma pathogenesis-related protein 1 (*GLIPR1*) and *C*ysteine-rich angiogenic inducer 61 (*CYR61*) were frequently lower expressed than their corresponding non-carcinogenic controls (figure 5I). This suggests that increased expression of these genes may contribute to a more physiologically normal cellular phenotype. To examine how the CCT3-*LINC00326* core genes were regulated, we determined DNA-binding motifs enriched in the promoter regions of the 70 commonly deregulated genes over 1000 random sequences (figure 5F, J, online supplemental figure S9f). The most significant motifs were recognised by CREM/CREB/ATF transcription factors (TFs) (figure 5J), which were entwined with regulation of lipid metabolism and general Pol II transcription (figure 5K). Moreover, ChIP-seq data in liver and liver cancer cell lines^23^ revealed direct binding of the CREM/CREB/ATF TFs to the promoters of the lipid metabolism related genes *EGR1*, *CYR61* and *GLIPR1* (figure 5L).

Thus, coordinated regulation of CREM/CREB/ATF TFs could lead to the observed expression changes of genes controlling lipid metabolism, hypoxia and angiogenesis.

### The CCT3-*LINC00326* network reduces tumour burden *in cellulo* and *in vivo*

**Figure 6:**
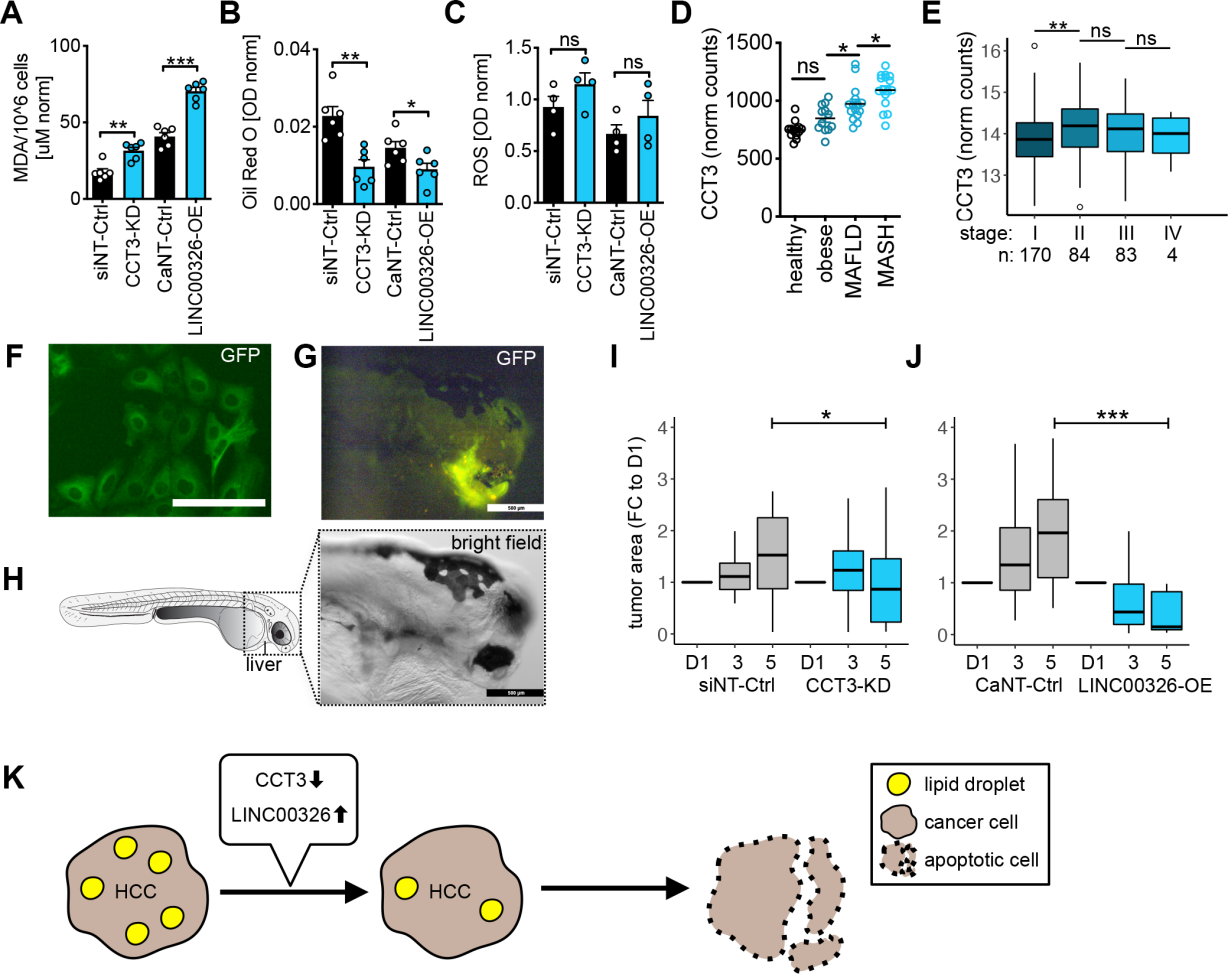
The CCT3-*LINC00326* network affects lipid metabolism and tumour growth *in vitro* and *in vivo*. (A–C) Bar graphs show comparison of (A) malondialdehyde (MDA) production (lipid degradation), (B) Oil Red O staining (lipid accumulation) and (C) ROS production of *CCT3*-KD or *LINC00326*-OE (blue) to the respective NT controls (black) 48 hours after transfection (n=4–6, mean, +SEM). Each biological replicate is displayed by circles. Statistics: paired two-tailed t-test, (D) Dot graph displays library size-normalised *CCT3* mRNA expression level in adult human individuals with normal and obese weight, MAFLD and MASH (n=2–8, mean). *LINC00326* was not assayed. Statistics: one-way ANOVA. (E) Boxplot of normalised *CCT3*-expression in the TCGA-LIHC cohort divided by main pathological cancer stage. Statistics: one-way ANOVA with Tukey Honest Significant Differences test. (F–H) Microscopy images of TUBULIN-GFP expressing Huh7 cells (F) *in vitro* (scale bar: 100µM) and (G–H) *in vivo* in zebrafish xenografts (scale bar: 500 µM).(I, J) Box plots show changes in tumour area in zebrafish xenografts after (I) *CCT3*-KD (n=20–21, mean, ±SEM) or (J*) LINC00326*-OE (n=21–28, mean, ±SEM). Individual zebrafish were followed over 5 days and tumour area is given relative fold change to day 1 (D1) after injection. Statistics: one-way ANOVA, *p<0.05, ***p<0.001. (K) Schematic model for CCT3-*LINC00326* regulation of lipid metabolism. Reducing *CCT3* or increasing *LINC00326* gene expression in liver cancer cells inhibits lipid accumulation and promotes lipid degradation (peroxidation). Due to the strong dependency of cancer cells towards high lipogenesis, this in turn slows down cancer growth and promotes cell death. *p<0.05, **p<0.01, ***p<0.001, MAFLD, metabolic-associated fatty liver disease; MASH, metabolic-associated steatohepatitis; NS, non-significant; ROS, reactive oxygen species.

Because our molecular data revealed that CCT3 and *LINC00326* affected lipid metabolism, we examined the cellular impact of this RBP-lincRNA interaction. By comparing *CCT3*-KD and *LINC00326*-OE to their respective controls, we measured a significant increase in lipid degradation (1.9 and 1.8 FC, respectively) (figure 6A), a significant decrease in lipid accumulation (−2.1 and −1.5 FC, respectively) (figure 6B) and elevated levels of reactive oxygen species (ROS) (both 1.3 FC) although not statistically significant (figure 6C). Since these assays confirmed that alteration of *CCT3* and *LINC00326* gene expression levels modulate regulation of lipid metabolism, we inspected publicly available data from patient liver biopsies with lipid metabolism disorders (GSE126848 and TCGA). We found an increase in *CCT3* gene expression, which correlated with the severity of metabolic-associated fatty liver disease and the pathological stage of HCC (figure 6D, E). This indicated that CCT3 functionality in cell lines can be recapitulated in the human body and is associated with one of the HCC aetiologies. *LINC00326* was not profiled in this study.

Because *LINC00326* had not been assessed *in vivo*, we performed cell line-derived xenograft experiments. We used human Huh7-GFP cells with reduced *CCT3* or elevated *LINC00326* gene expression, injected them into zebrafish embryos and monitored their cell growth over time. *CCT3*-KD and *LINC00326*-OE resulted in a significant suppression in tumour growth in comparison to the respective controls (figure 6F–J) confirming that low *CCT3* and high *LINC00326* gene expression reduced tumour burden.

In summary, our study demonstrated that functional lincRNAs can be identified by using an RBP-centric approach, through which we uncovered that the CCT3-*LINC00326* interaction regulates lipid metabolism in cancer cells. We show that modulation of lincRNA biogenesis via RBPs can alter cancer cell-specific activities, such as cancer cell survival and tumour growth (figure 6K).

## Discussion

Through advanced transcriptomic RBP-RNA profiling, RBP-RNA complex purification and functional screening (online supplemental table S1), the number of proteins with RNA-binding capacity has risen from a few hundreds to ~2300. *Bona fide* functions of canonical RBPs have been well characterised, and the role of non-canonical RBPs in RNA metabolism are beginning to be unravelled.^15^ By using an unbiased RBP-centric approach, we found that both canonical and non-canonical RBPs perturb liver cancer pathology by acting through an entangled network that required the involvement of lincRNAs. Phenotypic alterations were particularly severe after reducing gene expression of *CCT3* and *IGF2BP1*. Interestingly, the *CCT3*-KD influenced a large number of ncRNAs, especially lincRNAs and antisense RNAs. In contrast, the *IGF2BP1*-KD affected largely protein-coding transcripts, which has been observed previously.^29^ The preference towards specific RNA types could be explained through differences in RNA binding modalities. CCT3 is a non-canonical RBP without apparent RBDs, whereas IGF2BP1 represents a canonical RBP exerting its RNA binding activity through six RBDs.^11^ CCT3 together with seven other CCT subunits is known for forming a stoichiometrically even cytosolic chaperonin complex that ensures proper proteinfolding. Chaperonin-independent functions of each member have been speculated because of transcriptional and phenotypical differences after altering expression levels of individual CCT genes^30 31^ and disparities in protein abundance across intracellular compartments.^32^ Despite the high amino acid identity, each member evolved differences in protein regions that are essential for substrate specificity.^33^ Since the underlying genetic sequences of CCT genes are under purifying selection, new paralog-specific functions have been developing,^34^ perhaps even as a consequence of emerging new non-coding RNA substrates. It is therefore plausible that CCT3 functions as a non-canonical RBP independent of its role in the chaperonin complex. To gain a better understanding whether RBPs act through regulation of lincRNAs in HCC, we assayed four lincRNAs for which our data indicated a strong functional connection with RBPs. Three of the four lincRNAs (*LINC00326*, *LINC01419* and *LINC02119*) were previously annotated and one represented a novel lincRNA (*MSTRG.12891*). When altering the expression of these lincRNAs, we observed major transcriptional and phenotypical changes in HCC cells.

Across all assays performed, the CCT3-*LINC00326* interaction caused the most severe molecular and cellular effects. We detected elevated *CCT3* gene expression in HCC, which is in accordance to previous reports and underscores its prognostic value in HCC.^35 36^ Overall, *CCT3* was highly expressed in malignant cells when compared with various tissue types (figure 1, online supplemental figure S10a). In contrast, *LINC00326* abundance was low in tumour tissues but increased after the *CCT3-*KD in liver cancer cell lines (figure 3). Under normal physiological conditions, *LINC00326* was only detectable in testis (online supplemental figure S10b). Based on *LINC00326* gene expression patterns during spermatogenesis,^37^ *LINC00326* may function in cell proliferation and controlled apoptosis to eliminate irreparable damaged germ cells during development but the exact regulatory mechanisms remain to be determined. Interestingly, *LINC00326* gene expression is almost completely diminished in testicular cancers (online supplemental figure S10b) and gradually decreased with increased testicular cancer severity (online supplemental figure S10c). This implied that antiproliferative and proapoptotic properties of *LINC00326* (figure 4) peaks at the early stages of carcinogenesis. Besides, single-cell RNA-seq data from healthy testis showed high *CCT3* gene expression at early stages of spermatogenesis. Reduced *CCT3* gene expression at a later developmental stageincreased *LINC00326* levels (online supplemental figure S10d–f). This temporal expression pattern in testis is in accordance with our *CCT3*-KD and suggests that high levels of CCT3 suppress *LINC00326*.

Reducing gene expression from a high to a moderate level has smaller effects than increasing gene expression from a low to a high level.^38^ Accordingly, KD of the highly expressed *CCT3* caused a small increase of *LINC00326* transcript abundance, while CRISPRa-mediated OE substantially stimulated *LINC00326* gene expression and could explain why more genes were affected by *LINC00326*-OE than *CCT3*-KD in liver cancer cells. Inspection of genes overlapping both perturbations revealed an involvement in controlling lipid metabolism pathways. Our comprehensive cell-based assays confirmed that *LINC00326*-OE and *CCT3*-KD led to a decrease in lipid accumulation and increase in peroxidation. Our findings support the increasing recognition of lipid metabolism as cancer confounder.^39 40^ Response to hypoxia and oxygen levels as well as angiogenesis were additional commonly induced pathways, which are consistent with prior reports ascribing atypic hypoxic and angiogenic conditions to tumours.^41^ These pathways are linked mechanistically whereby a hypoxic condition leads to an excess of NADPH, which is used for lipogenesis, and has been proposed to maintain a balanced redox environment.^42^ Accordingly, we also observed a trend towards increased ROS-production after *CCT3*-KD or *LINC00326*-OE. Taken together, our data supported that the CCT3-*LINC00326* network plays a vital role in liver cancer pathology via perturbating lipid metabolism.

Inspection of the promoter regions of deregulated genes emerging after altering *CCT3* and *LINC00326* gene expression revealed a common set of enriched transcription factors (TFs) that acted in a coordinated manner (figure 5K). Our network analysis showed that the TFs CREM, CREB and ATF were not only linked to the Pol II machinery but also to genes that regulate lipid metabolism. Furthermore, we found experimental evidence of TF-binding to promoters of lipid metabolic genes (figure 5L). For instance, we confirmed strong CREM/CREB1-binding to the *EGR1* promoter. EGR1 is in itself a TF, and accumulating evidence substantiates its tumour suppressing role in HCC.^43^ Loss of EGR1 and tumour development are connected through oncogenic RAS-PI3K signalling^44^, which is also a top GO term that we identified after *LINC00326*-OE (online supplemental figure S7). *CCT3*-KD and *LINC00326*-OE did not lead to upregulation of these TF genes but TF activity can still be altered. Previous studies described that ATF2^45^ and JUN^46^ proteins interact with CCT3. It is therefore plausible that after upregulation of CCT3 in HCC, CCT3 binds to TFs (including ATF2), sequesters and thereby prevents TFs from binding to promoter regions of genes involved in lipid metabolism and *LINC00326* (online supplemental figure S11a). Our RIP-qPCR assay confirmed the interaction of CCT3 and *LINC00326* (figure 5A). When *LINC00326* binds to CCT3, it may impede CCT3’s confinement of ATF2, thereby releasing ATF2 from its inactive state and thus allowing transcription of lipid metabolism genes and *LINC00326* itself (online supplemental figure S11b). Further studies will shed more light on the regulatory intricacies of the proposed CCT3-*LINC00326* network.

Most RBPs were identified in more than one recent study (average: 6.5, median: 5) (online supplemental table S1) and many have not yet been validated. With 2282 detectable RBPs in HCC, including 959 non-canonical, the future challenge is to establish the exact functional links between RBP and RNA. Nevertheless, by using an RBP-centric approach, we have identified prognostically relevant RBPs and lincRNAs with major functional molecular and cellular roles in HCC. The combination of loss-of-function and gain-of-function *in cellulo* and *in vivo* experiments allowed us to construct networks that regulate oncogenic lipid metabolism, impair cellular energy consumption and increase intracellular oxidative stress. Given that the lincRNAs investigated in this study are barely expressed under normal physiological conditions, we speculate that they act as an inhibitor to prevent cellular transformation of healthy hepatocytes into malignant cells. As such, they could represent novel markers for liver pathologies and molecular targets for future HCC treatment approaches.

## Materials and methods

Cell-based and molecular assays as well as xenograft experiments are described in the online supplemental materials and methods.

10.1136/gutjnl-2021-325109.supp2 Supplementary data

Supplementary tables and microscopic imaging files are accessible via Figshare: https://figshare.com/s/2c05765158269b3b4ff2, https://figshare.com/s/a83dbee52555e922ca8d and https://figshare.com/s/08b0f84f2ea241b03c8d.

Datasets generated in this study are deposited under ArrayExpress accessions E-MTAB-8915, E-MTAB-9587 and E-MTAB-9586.

Scripts used for bioinformatics analyses are available on Github: https://githubcom/jonasns/LiveRNome.

### Patient material

Patients (75% men and 25% women) taking part in this study had HCC from Hepatitis B virus infection, non-alcoholic fatty liver disease, alcoholic steatohepatitis, hereditary haemochromatosis and other HCC-triggering conditions. See patient information on ArrayExpress E-MTAB-8915. It was neither possible nor appropriate to involve patients or the public in the design, conduct, reporting or dissemination plans of our research.

10.1136/gutjnl-2021-325109.supp3 Supplementary data

## Data Availability

Data are available in a public, open access repository. All data relevant to the study are included in the article or uploaded as supplementary information. All data generated or analysed during this study are included in the article. Supplementary tables and microscopic imaging files are accessible via Figshare: https://figshare.com/s/2c05765158269b3b4ff2, https://figshare.com/s/a83dbee52555e922ca8d and https://figshare.com/s/08b0f84f2ea241b03c8d. Original sequencing data are available from ArrayExpress under accession numbers: E-MTAB-8915, E-MTAB-9587 and E-MTAB-9586. Scripts used for bioinformatics analyses are available on Github: https://github.com/jonasns/LiveRNome. Raw data not available in the online supplemental tables can be found in supplemental data 1. Additionally, all relevant data are available from the authors on request.
