# Supplementary material for: CCT3-LINC00326 axis regulates hepatocarcinogenic lipid metabolism
Source: Gut. 2022 Jan 12;71(10):2081–92. doi: 10.1136/gutjnl-2021-325109 (PMC9484377; doi:10.1136/gutjnl-2021-325109)
Supplement: Supplementary data [file gutjnl-2021-325109supp001.pdf]

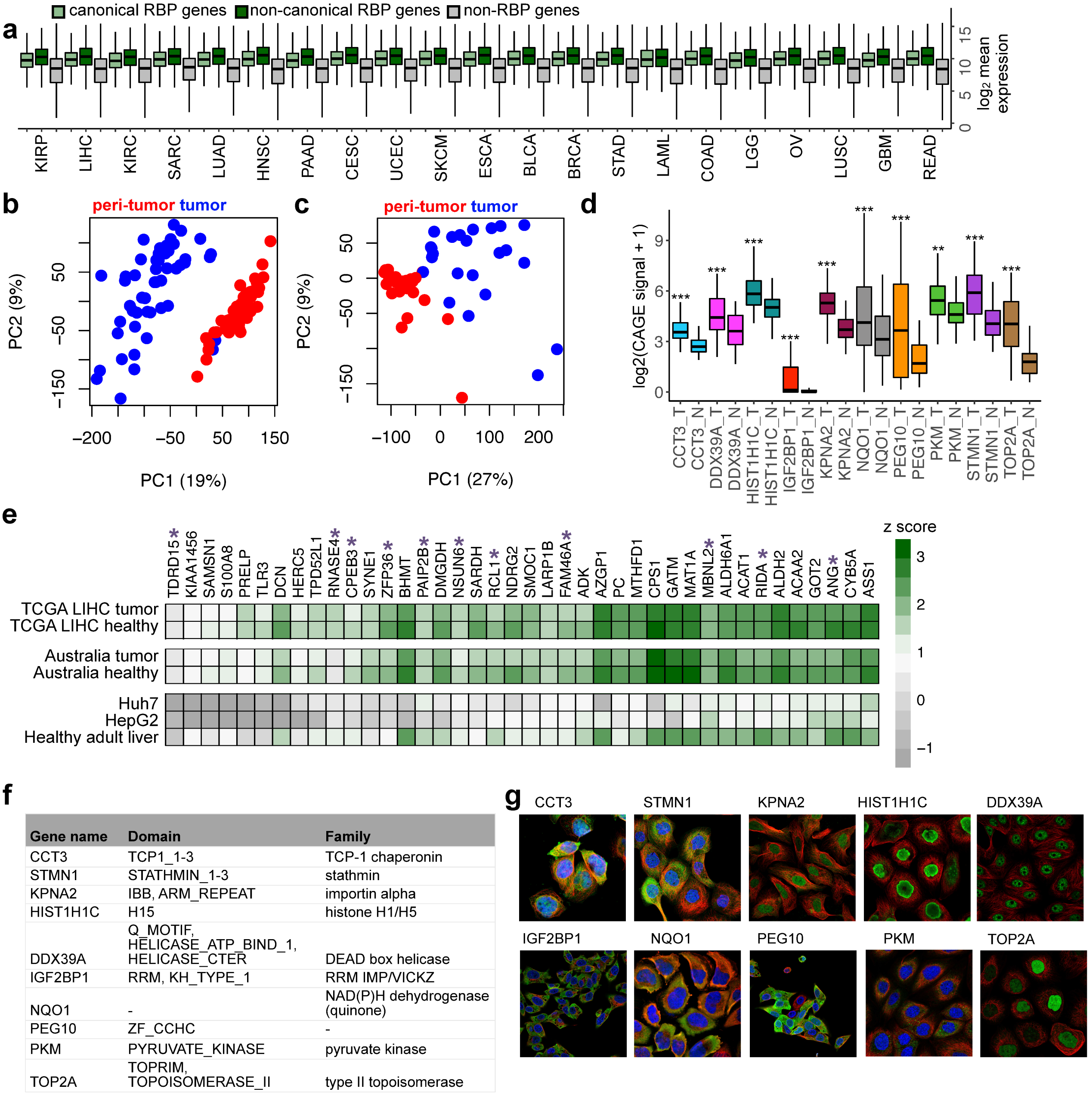

Gene name	Domain	Family
CCT3	TCP1_1-3	TCP-1 chaperonin
STMN1	STATHMIN_1-3	stathmin
KPNA2	IBB, ARM_REPEAT	importin alpha
HIST1H1C	H15	histone H1/H5
DDX39A	Q_MOTIF, HELICASE_ATP_BIND_1, HELICASE_CTER	DEAD box helicase
IGF2BP1	RRM, KH_TYPE_1	RRM IMP/VICKZ
NQO1	-	NAD(P)H dehydrogenase (quinone)
PEG10	ZF_CCHC	-
PKM	PYRUVATE_KINASE	pyruvate kinase
TOP2A	TOPRIM, TOPOISOMERASE_II	type II topoisomerase

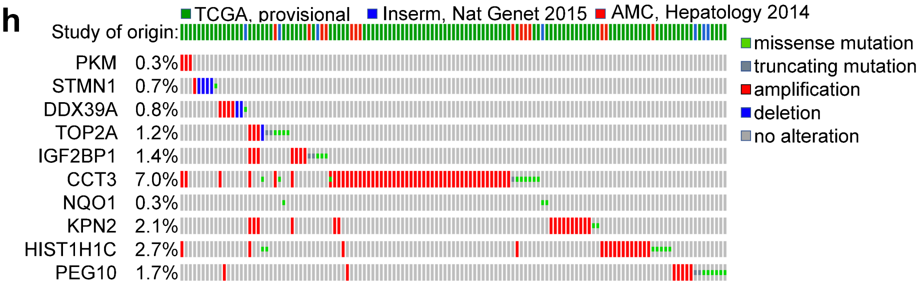

Fig. S1: Molecular characteristics of RBPs in HCC.
a, Boxplots of gene expression level of protein-coding genes grouped into canonical-RBP-genes (light-green), non-canonical-RBP-genes (dark-green), and non-RBP-genes (grey). Each column represents a different cancer type defined by The Cancer Genome Atlas (TCGA). **b-c,** Factorial map of the principal components (PC) analysis separates size factor and $\log_2$ normalized gene expression levels of the (**b**) TCGA LIHC (50 patients) and (**c**) Australia HCC (24 patients) cohort into tumor (blue) and peritumor (red) patient samples. The proportion of variance explained by each principal component is indicated in parenthesis. **d,** Box plots represent the expression levels of the ten selected RBP genes in the FANTOM HCC cohort (50 patients) (Hashimoto *et al.*, Genome Res., 2015) . Hinges correspond to the first and third quartiles, and whiskers correspond to the 1.5-times interquartile range. T: tumor; N: peritumor tissue. Statistics: paired two-tailed t-test, **p<0.01, ***p<0.001. **e,** Heatmap displays changes in expression level for commonly downregulated RBP genes in HCC as highlighted in (Fig. 1e). Expression level is sorted by average tumor z-score from right to left. Color gradient indicates z-score differences (green: high; grey: low). **f,** Table displays protein domains and family classification of the ten selected RBPs according to ExPASy (https://prosite.expasy.org). **g,** Microscopy images showing cellular location of immunofluorescently labeled RBPs (blue: DAPI staining of the nucleus, red: microtubules, green: RBP) in human cells (HepG2, A-431, U-2 OS, U-251) retrieved from the Human Protein Atlas (https://www.proteinatlas.org). **h,** Mutational status of the 10 selected RBP genes from three independent cohort studies (top panel, green: CGA, blue: INSERM, green: AMC) deposited in cBioPortal (http://www.cbioportal.org) shows proportional frequency of missense mutations (green), truncations (purple), amplifications (red), deletions (blue) and no alterations (grey). The percentage indicates the total number of alterations in all patients (n=865).

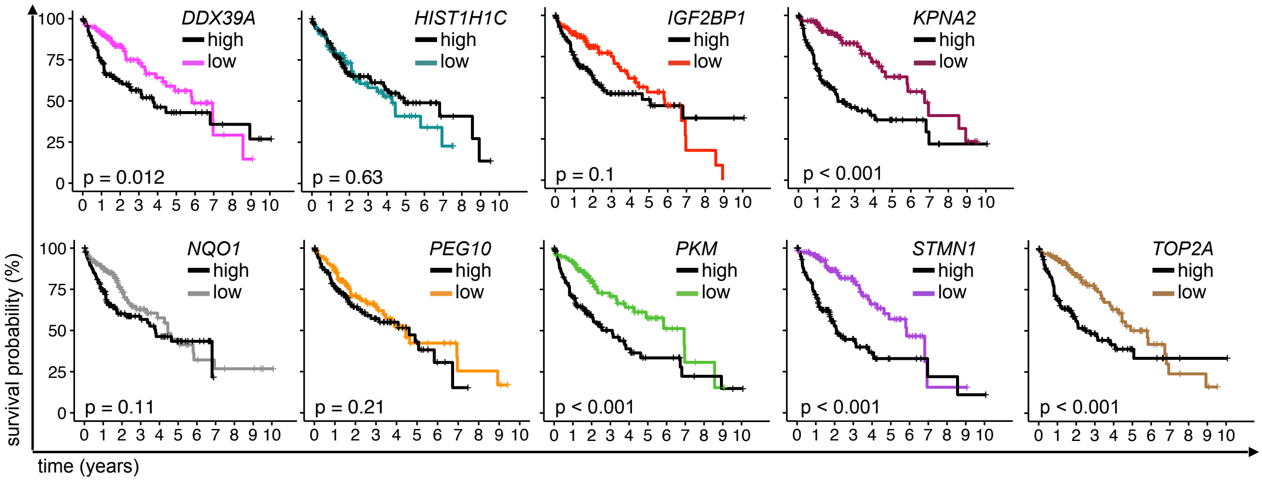

Fig. S2: Survival analysis of the TCGA-LIHC cohort.

Kaplan-Meier plots show the association of RBP gene expression level and ten-year survival within the top and bottom tercile of 377 TCGA-LIHC (black: high and colored: low RBP gene expression levels, color code: DDX39A (magenta), HIST1H1C (turquoise), IGF2BP2 (red), *KPNA2* (plum), NQO1 (grey), PEG10 (yellow), PKM (green), STMN1 (purple) and TOP2A (brown)). Statistics: log-rank (Mantel-Cox).

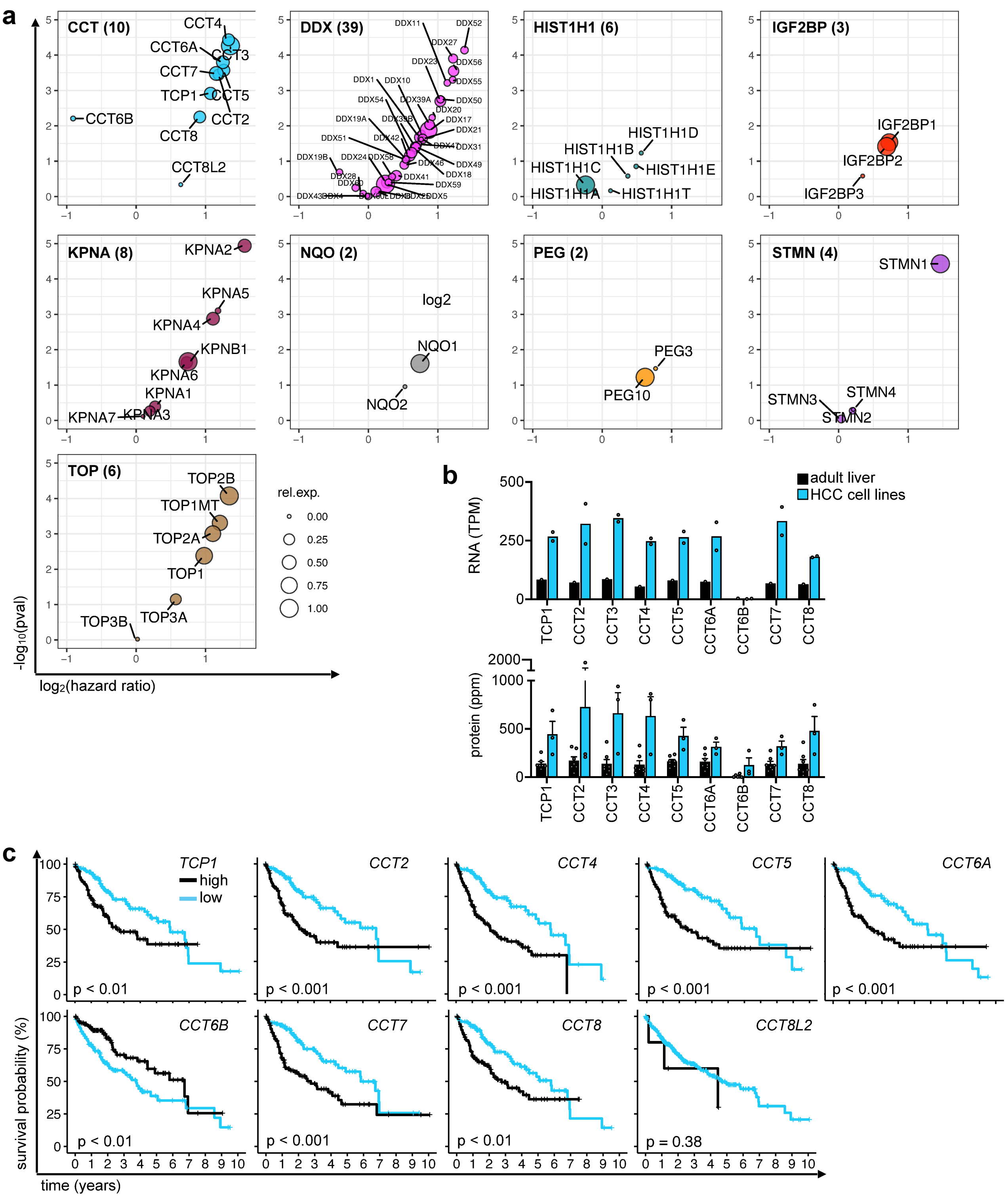

Fig. S3: Estimation of hazard ratio and HCC patient survival based on RBP gene expression.
a, Volcano plots display statistical significance (FDR<0.05, likelihood ratio test) and hazard ratio comparing the top and bottom terciles of RBP genes grouped by family. Numbers in brackets after the name of the gene family represent the total number of family members. PKM does not have any family members and is hence not displayed. The size of the circle represents the relative gene expression level of each gene per family (broad: high, narrow: low). **b**, Barplots demonstrate RNA (top) and protein (bottom) expression of the CCT3 family members. Data were obtained from Rudolph *et al.*, PLoS Genet, 2016; PAXdb v. 4.1 pax-db.org, accessed on 2021-03-24). **c,** Kaplan-Meier plots shows the effect of CCT3-family members expression on ten-year survival within the top and bottom tercile of the TCGA-LIHC cohort (377 patients). Statistics: log-rank (Mantel-Cox).

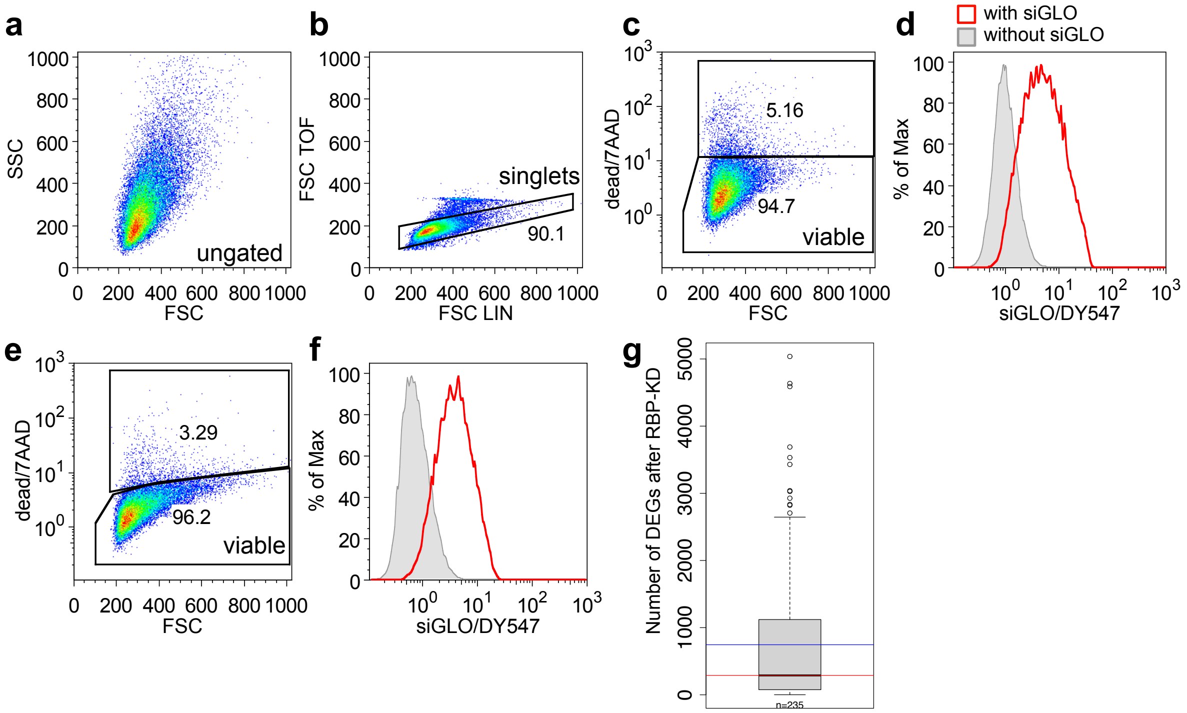

Fig. S4: siRNA transfection efficiency and RBP-KD perturbation effects in human HCC cells

a-f, Flow cytometry plots displaying gating strategy and transfection efficiency of siRNA (here siGLO) in **a-d** Huh7 and **e-f** HepG2 cells after electroporation. The completely shifted flow cytometry histogram indicates 100% transfection efficiency. Cell dead marker 7AAD was used to count dead and viable cells (shown as percentage). **g**, Boxplot showing the number of significantly differentially expressed genes (DEGs) upon RBP-KD in the ENCODE project database. Y axis represents number of DEGs; n represents the number of KD experiments; the box encloses all the data points between the upper and lower quartile; whiskers are drawn to the furthest data point falling within 1.5-times the interquartile range. Points represent outliers. The red line is median of the distribution and the blue line the mean (both median and mean are annotated with their respective values). Data is from (Van Nostrand *et al.*, Nature, 2020; Davis *et al.*, Nucleic Acids Res., 2018).

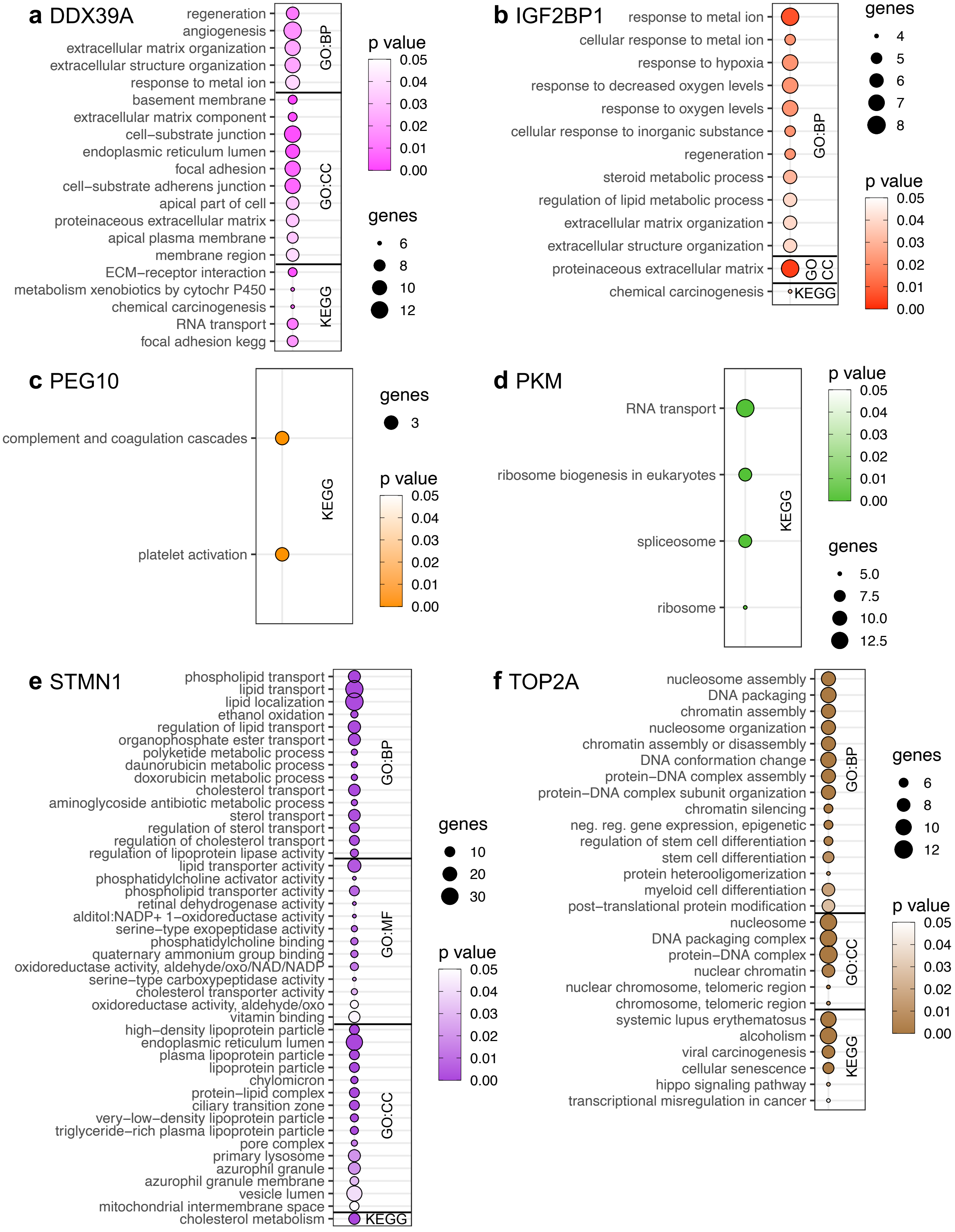

Fig. S5: Gene Ontology (GO) and KEGG analyses of deregulated genes upon RBP-KD
a-f, Circle plot shows GO term and KEGG pathway enrichment analysis of deregulated genes (FDR<0.05) after the siRNA-mediated KD of RBP **a**, DDX39A; **b**, IGF2BP1; **c**, PEG10; **d**, PKM; **e**, STMN1 and **f,** TOP2A. No significant pathways were found for genes deregulated upon KD of HIST1H1C, KPNA2, and NQO1. The diameter of the circles corresponds to the number of genes in each GO or KEGG term and the color code represents varying degrees of significance for the respective RBP-KD (white: high and colored: low adjusted p-value). BP, biological process; MF, molecular function; CC, cellular compartment.

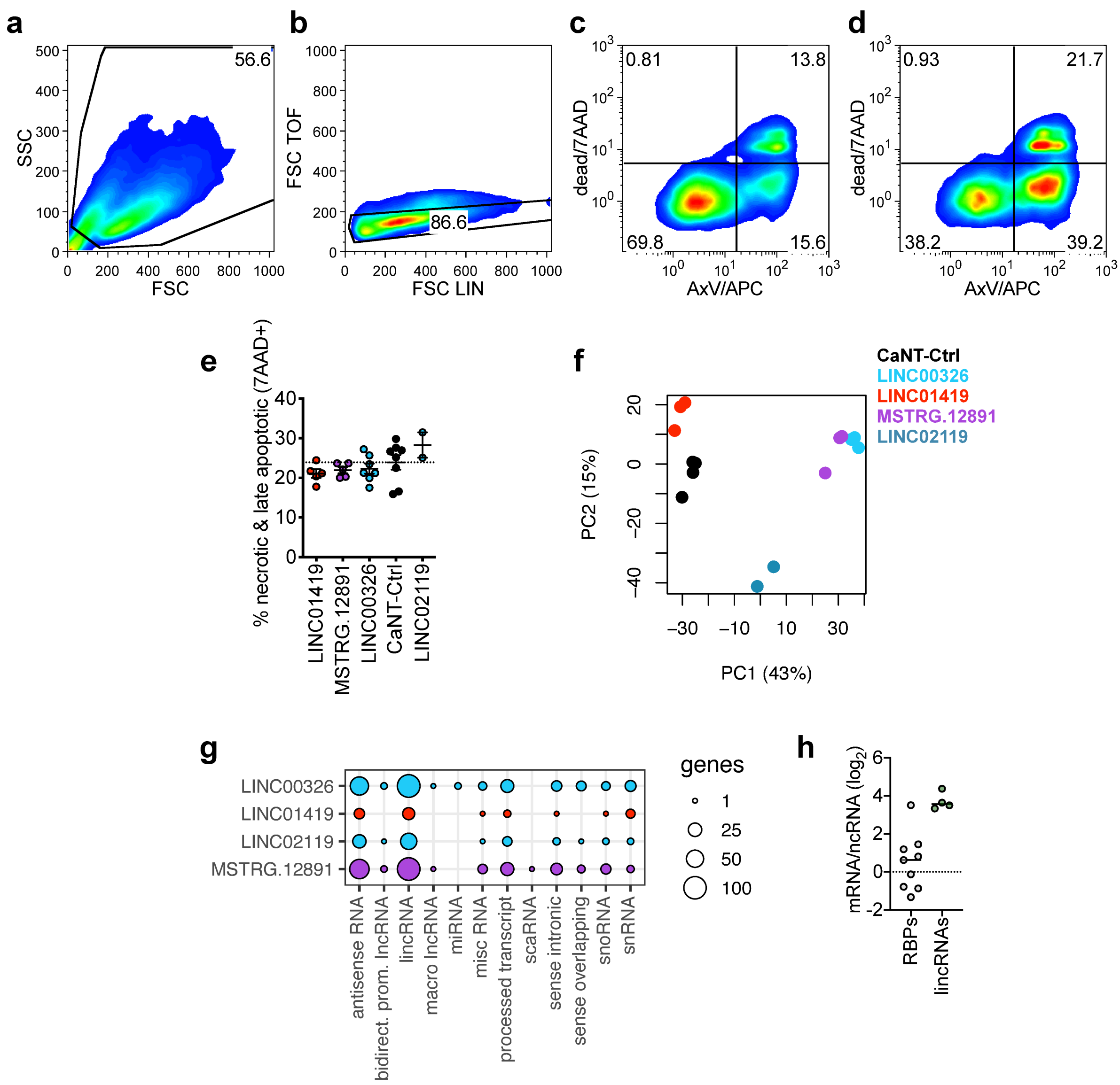

Fig. S6: Cellular and molecular phenotype assessment upon lincRNA-OE

a-d, Flow cytometry gating strategy for assaying early apoptosis (Annexin V, AxV) and late apoptosis/necrosis (7AAD). **e**, dot plot displays the percentage of necrotic and late apoptotic (7AAD+ cells assayed by flow cytometry) in Huh7 and HepG2 cells after CRISPRa-mediated lincRNA-OE after five days (n=2-8, mean, +/-SEM). The color-code links the lincRNA to the respective RBP-KD experiment through which it was identified (blue: CCT3, red: IGF2BP1, purple: STMN1). **f,** Factorial map of the principal components (PC) analysis separates size factor and $\log_2$ normalized gene expression levels of the CRISPRa-mediated lincRNA-OE. The proportion of variance explained by each principal component is indicated in parenthesis. **g,** Circle plots display number of genes per non-coding RNA biotype affected by the CRISPRa-mediated lincRNA-OE. The diameter of the circles corresponds to the number of genes in each category. **h,** Dot plot of mRNA/lincRNA ratio after RBP-KD (n=10) or lincRNA-OE (n=4). The mean ratio is indicated by a black line.

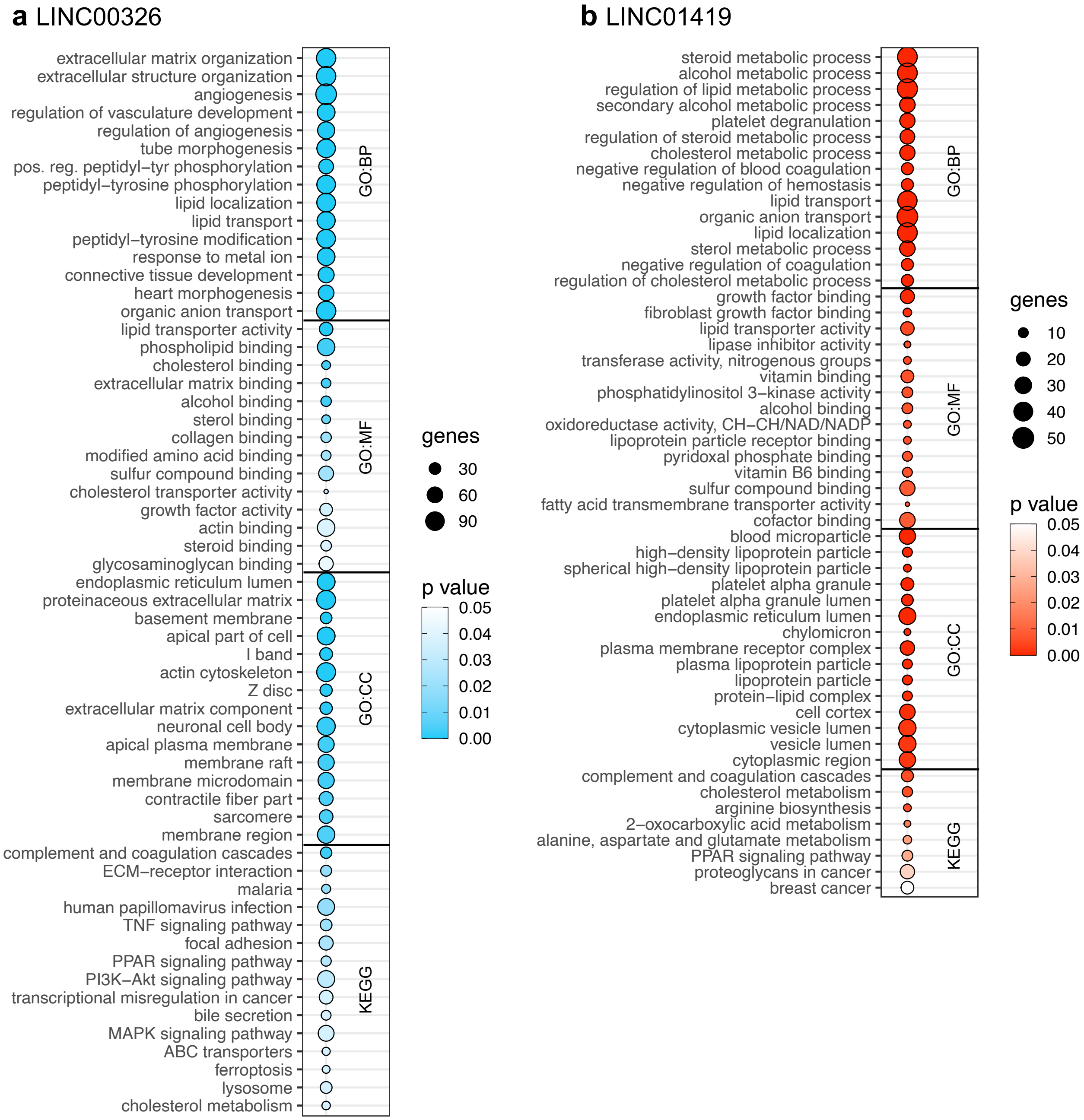

Fig. S7: Gene Ontology (GO) and KEGG analyses of deregulated genes upon OE

a-d, Circle plot shows GO term and KEGG pathway enrichment analysis of deregulated genes (FDR<0.01) after the CRISPRa-mediated lincRNA-OE **a**, *LINC00326*; **b**, *LINC01419*; **c**, *LINC02119* and **d**, *MSTR.12891*. The size of the circles indicates number of genes in each GO or KEGG term and the color represents varying degrees of significance for the respective lincRNA-OE experiment (white: high and colored: low adjusted p-value). The color-code links the lincRNA to the respective RBP-KD experiment through which it was identified (blue: CCT3, red: IGF2BP1, purple: STMN1). A maximum of 15 terms are shown in each GO or KEGG term (full list is available in Table S13). BP, biological process; MF, molecular function; CC, cellular compartment. Figure continues on the next page.

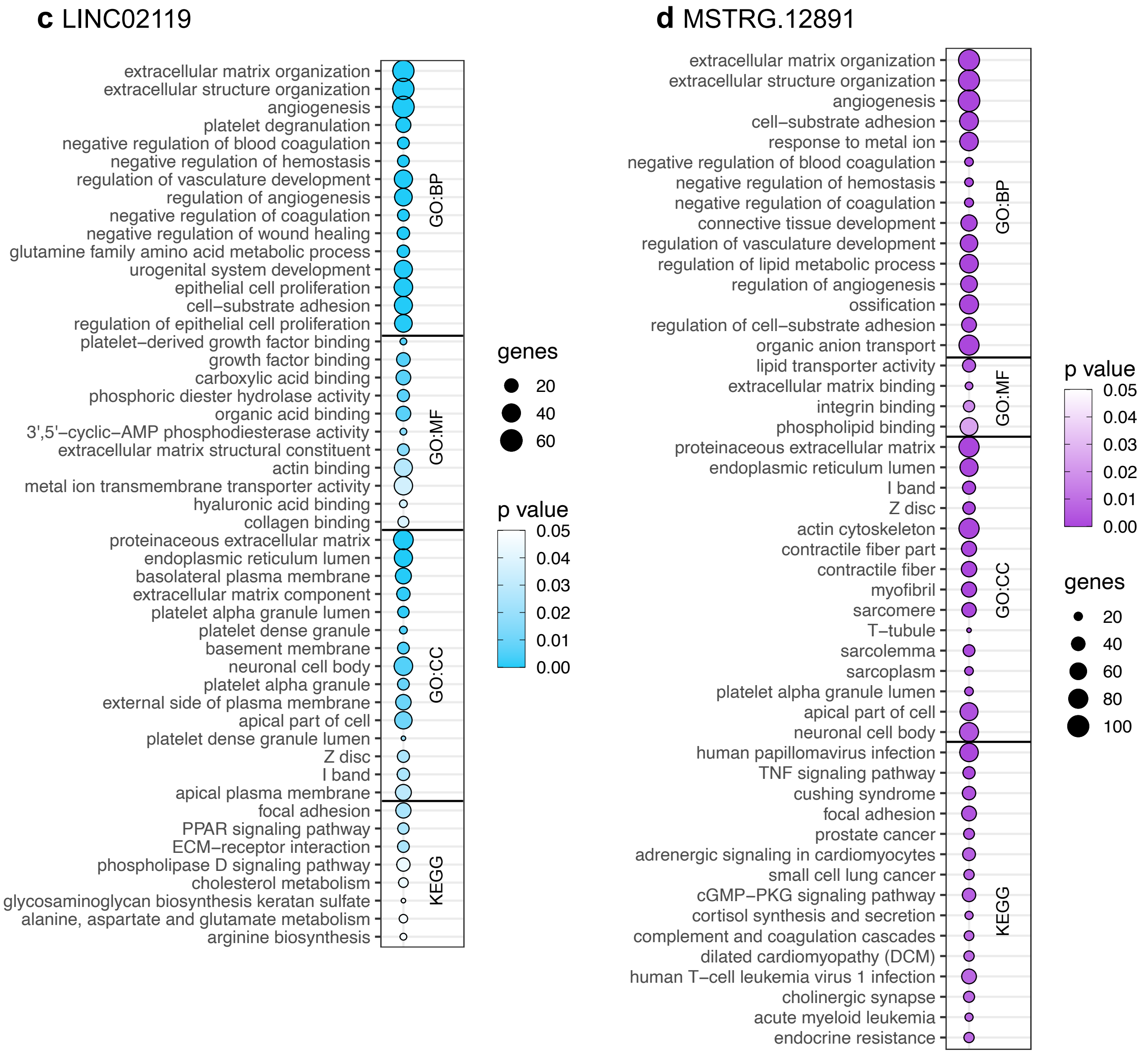
c LINC02119
extracellular matrix organization
extracellular structure organization
angiogenesis
platelet degranulation
negative regulation of blood coagulation
negative regulation of hemostasis
regulation of vasculature development
regulation of angiogenesis
negative regulation of coagulation
negative regulation of wound healing
glutamine family amino acid metabolic process
urogenital system development
epithelial cell proliferation
cell−substrate adhesion
regulation of epithelial cell proliferation
GO:BP
platelet−derived growth factor binding
growth factor binding
carboxylic acid binding
phosphoric diester hydrolase activity
organic acid binding
3',5'−cyclic−AMP phosphodiesterase activity
extracellular matrix structural constituent
actin binding
metal ion transmembrane transporter activity
hyaluronic acid binding
collagen binding
GO:MF
proteinaceous extracellular matrix
endoplasmic reticulum lumen
basolateral plasma membrane
extracellular matrix component
platelet alpha granule lumen
platelet dense granule
basement membrane
neuronal cell body
platelet alpha granule
external side of plasma membrane
apical part of cell
platelet dense granule lumen
Z disc
I band
apical plasma membrane
GO:CC
focal adhesion
PPAR signaling pathway
ECM−receptor interaction
phospholipase D signaling pathway
cholesterol metabolism
glycosaminoglycan biosynthesis keratan sulfate
alanine, aspartate and glutamate metabolism
arginine biosynthesis
KEGG
genes
20
40
60
p value
0.05
0.04
0.03
0.02
0.01
0.00
d MSTRG.12891
extracellular matrix organization
extracellular structure organization
angiogenesis
cell−substrate adhesion
response to metal ion
negative regulation of blood coagulation
negative regulation of hemostasis
negative regulation of coagulation
connective tissue development
regulation of vasculature development
regulation of lipid metabolic process
regulation of angiogenesis
ossification
regulation of cell−substrate adhesion
organic anion transport
GO:BP
lipid transporter activity
extracellular matrix binding
integrin binding
phospholipid binding
GO:MF
proteinaceous extracellular matrix
endoplasmic reticulum lumen
I band
Z disc
actin cytoskeleton
contractile fiber part
contractile fiber
myofibril
sarcomere
T−tubule
sarcolemma
sarcoplasm
platelet alpha granule lumen
apical part of cell
neuronal cell body
GO:CC
human papillomavirus infection
TNF signaling pathway
cushing syndrome
focal adhesion
prostate cancer
adrenergic signaling in cardiomyocytes
small cell lung cancer
cGMP−PKG signaling pathway
cortisol synthesis and secretion
complement and coagulation cascades
dilated cardiomyopathy (DCM)
human T−cell leukemia virus 1 infection
cholinergic synapse
acute myeloid leukemia
endocrine resistance
KEGG
p value
0.05
0.04
0.03
0.02
0.01
0.00
genes
20
40
60
80
100

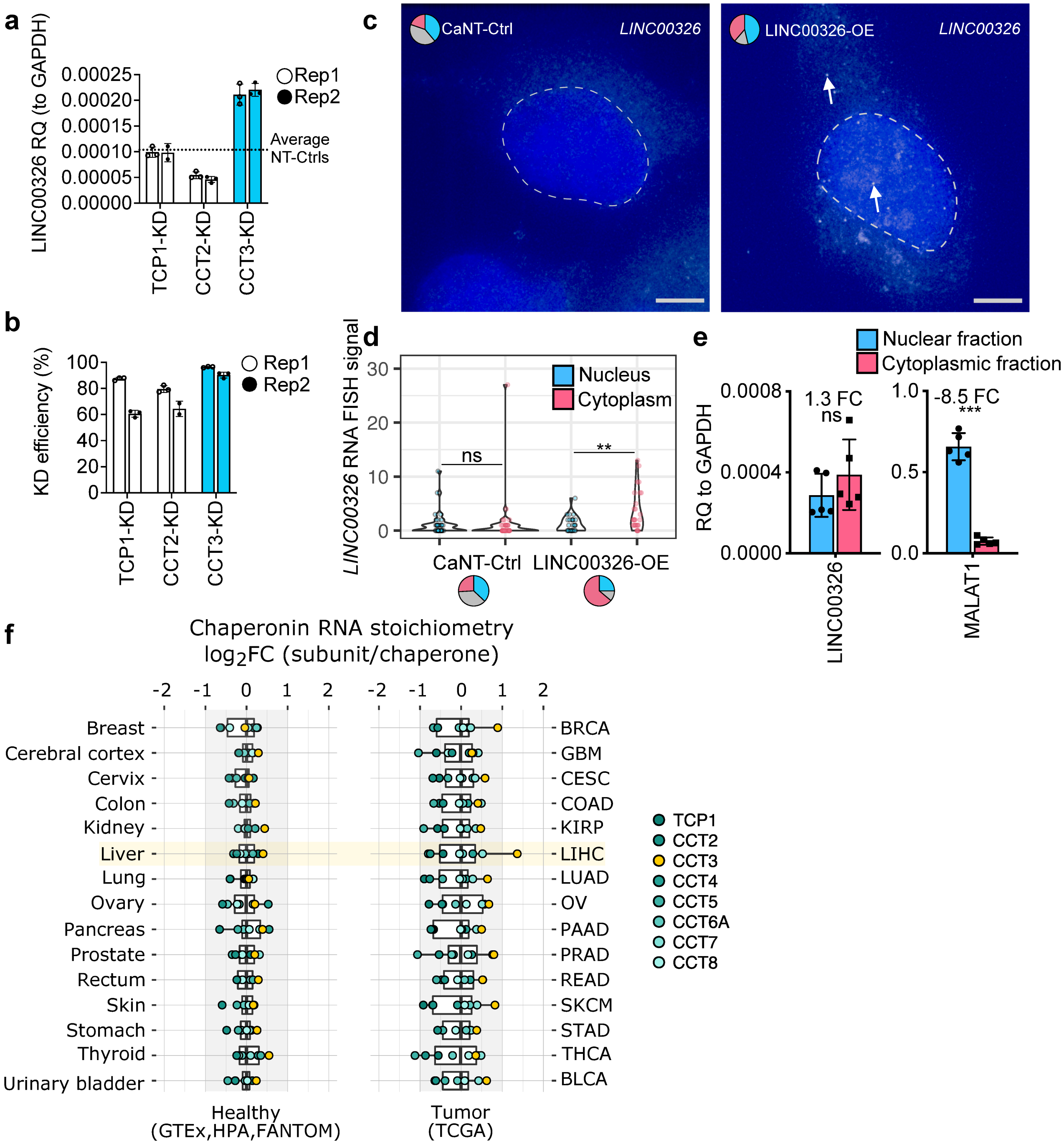

Fig. S8: CCT3 works in a chaperoning-independent manner to interact with LINC00326.
a, Bar graph shows the gene expression levels of *LINC00326* after CCT-family member KD. **b**, Bar graph shows KD-efficiency of samples in a. **c**, Microscopic images of single-molecule RNA FISH using exonic probes for *LINC00326* (white dots and arrows) in CaNT-Ctrl (left) and *LINC00326*-OE (right) Huh7 HCC cells. DAPI (blue) marks the nucleus. Pie chart represents the fraction of signals in the nucleus (blue) or cytoplasm (pink) in cells, or cells without any signal (grey). Scale bar: 5 µm. **d**, Violin plots show quantification of *LINC00326* RNA FISH signal localization in HepG2 cells (n = 27-35). The pie chart represents the fraction of signal in the nucleus (blue) or cytoplasm (pink). Statistics: paired two-tailed t-test, ** p<0.01, ns: not significant. **e**, Bar graphs show gene expression patterns of *LINC00326* and *MALAT1* in separate cellular fractions of *LINC00326*-OE HCC cells. Quantification was performed by qPCR and gene expression values were normalized to *GAPDH*. Statistical significance and absolute fold change (FC) are displayed above the graphs. Statistics: paired two-tailed t-test, *** p<0.001. (n = 5). **f**, Box plots shows RNA stoichiometry data for subunits of the CCT chaperonin complex. The x axis value is computed as the fold change between the median gene expression level of the subunit and the median gene expression level of all subunits. The left boxes show healthy tissues and the right boxes show corresponding tissue tumors. Data were retrieved from the Human Protein Atlas (v. 20.1, https://www.proteinatlas.org).

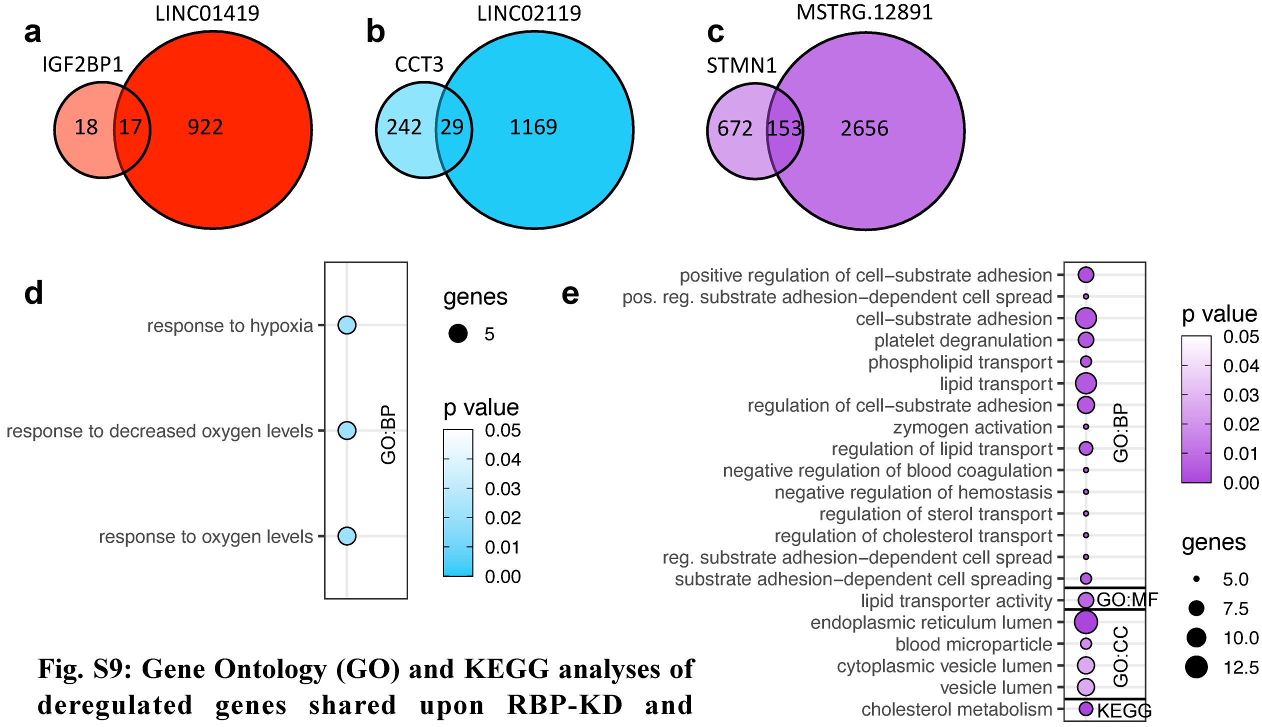

Fig. S9: Gene Ontology (GO) and KEGG analyses of deregulated genes shared upon RBP-KD and lincRNA-OE

a-c, Two-way Venn diagrams intersect the number of deregulated genes in the RBP-KD and lincRNA-OE. **a,** *IGF2BP1*-KD and *LINC01419*-OE, **b,** *CCT3*-KD and *LINC2119*-OE, **c,** *STMN1*-KD and *MSTR.12891*-OE. **d-e,** GO and KEGG pathway analysis of overlapping genes from (b) *CCT3*-KD and *LINC2119*-OE and (c) *STMN1*-KD and *MSTR.12891*-OE. No significant pathways were found for genes deregulated upon *IGF2BP1*-KD and *LINC01419*-OE. The diameter of the circles corresponds to the number of genes in each GO or KEGG term and the color code represents varying degrees of significance for the respective RBP-KD and lincRNA-OE (white: high and colored: low adjusted p-value). BP, biological process; MF, molecular function; CC, cellular compartment. A maximum of 15 terms are shown in each category (full list is available in Table S13). **f**, Identified motifs for each TF in **Fig. 5f**.

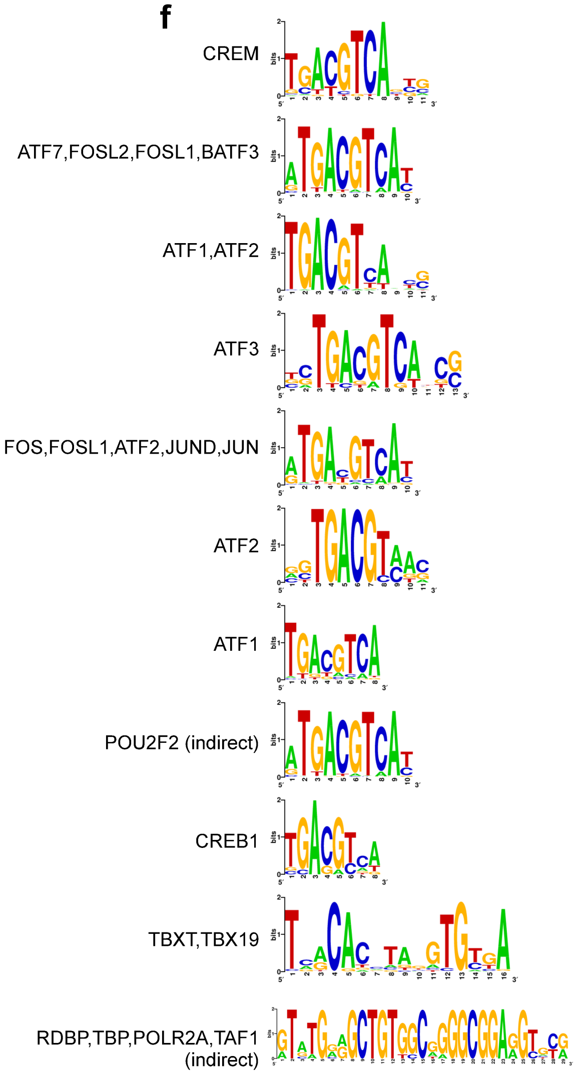

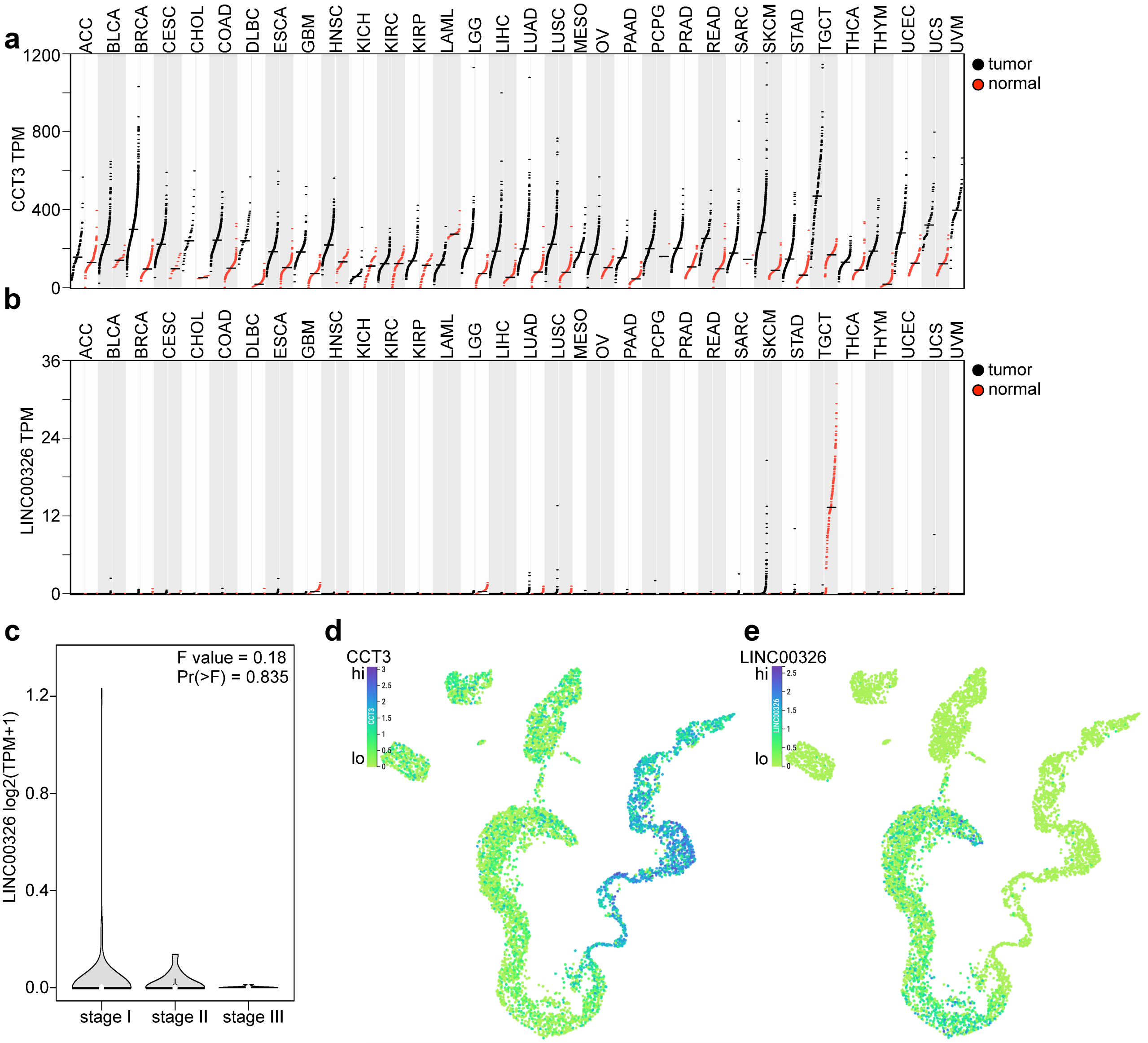

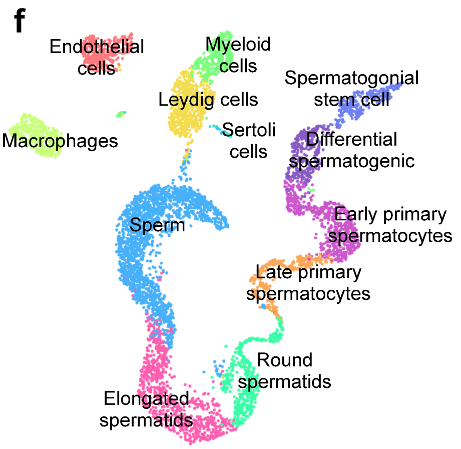

Fig. S10: LINC00326 is highly expressed in healthy testis and diminished upon cancer progression

a-b, Dot plot demonstrating the expression level in TPM of (a) CCT3 and (b) LINC00326 across different tissues from the TCGA and GTEx dataset. ACC (n=77-128 (tumor-normal)), BLCA (n=404-28), BRCA (n=1085-291), CESC (n=306-13), CHOL (n=36-9), COAD (n=275-349), DLBC (n=47-337), ESCA (n=182-286), GBM (n=163-207), HNSC (n=519-44), KICH (n=66-53), KIRC (n=523-100), KIRP (n=286-60), LAML (n=173-70), LGG (n=518-207), LIHC (n=369-160), LUAD (n=483-347), LUSC (n=486-338), MESO (n=87-0), OV (n=426-88), PAAD (n=179-171), PCPG (n=182-3), PRAD (n=492-152), READ (n=92-318), SARC (n=262-2), SKCM (n=460-558), STAD (n=408-211), TGCT (n=137-165), THCA (n=512-337), THYM (n=118-339), UCEC (n=174-91), UCS (n=57-78), UVM (n=79-0). **c**, LINC00326 expression in tumor biopsies from testis cancer patients divided by cancer stage. **d-e**, UMAP of single cell RNA expression of (d) *CCT3* (e) *LINC00326* in (f) human testis cells. a-c data are from: GEPIA (Tang *et al.* Nucleic Acids Res., 2017), and d-e are from Guo *et* al. Cell Res., 2018, visualised at covid19cellatlas.org (accessed on 21.3.21). ACC: adrenocortical carcinoma, BLCA: bladder urothelial carcinoma, BRCA: breast invasive carcinoma, CESC: cervical squamous cell carcinoma and endocervical adenocarcinoma, CHOL: cholangiocarcinoma, COAD: colon adenocarcinoma, DLBC: lymphoid neoplasm diffuse large B-cell lymphoma, ESCA: esophageal carcinoma, GBM: glioblastoma multiforme, HNSC: head and neck squamous cell carcinoma, KICH: kidney chromophobe, KIRC: kidney renal clear cell carcinoma, KIRP: kidney renal papillary cell carcinoma, LAML: acute myeloid leukemia, LGG: brain lower grade glioma, LIHC: liver hepatocellular carcinoma, LUAD: lung adenocarcinoma, LUSC: lung squamous cell carcinoma, MESO: mesothelioma, OV: ovarian serous cystadenocarcinoma, PAAD: pancreatic adenocarcinoma, PCPG: pheochromocytoma and paraganglioma, PRAD: prostate adenocarcinoma, READ: rectum adenocarcinoma, SARC: sarcoma, SKCM: skin cutaneous melanoma, STAD: stomach adenocarcinoma, TGCT: testicular germ cell tumors, THCA: thyroid carcinoma, THYM: thymoma, UCEC: uterine corpus endometrial carcinoma, UCS: uterine carcinosarcoma, UVM: uveal melanoma.

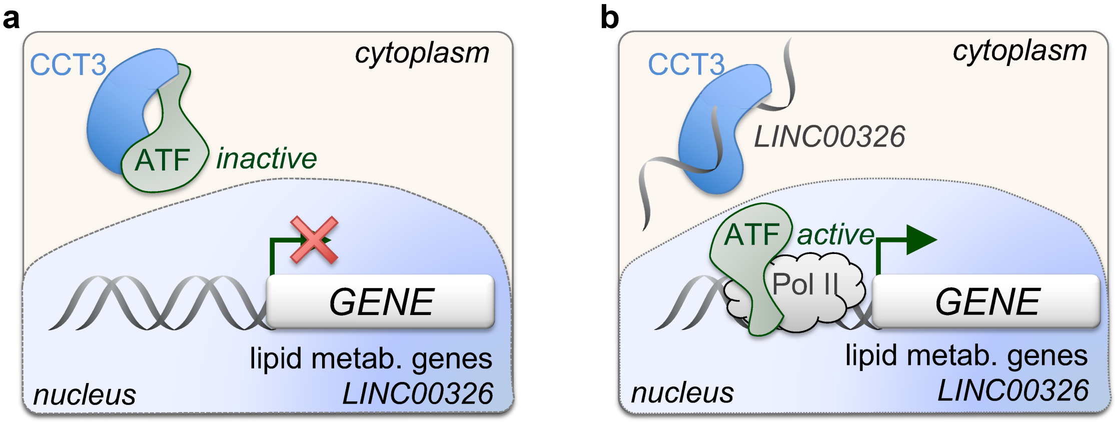

Fig. S11: Molecular model for LINC00326 regulating lipid metabolism-associated gene expression

a, In HCC, CCT3 moonlights as an RBP, perhaps due to increased gene expression of CCT3. CCT3 binds to transcription factor ATF mRNA or forms a heteromeric protein complex. This causes that CCT3 sequester away ATF from initiating transcription of genes associated with lipid metabolism or *LINC00326*. **b,** when expressed, *LINC00326* RNA interacts with CCT3. This consequently limits the binding of CCT3 and ATF protein, thus releasing ATF and allowing transcription of lipid metabolism genes and *LINC00326* itself. A similar molecular mechanism occurs when CCT3 is reduced, thereby enabling expression of *LINC00326*.
