# Supplementary material for: CCT3-LINC00326 axis regulates hepatocarcinogenic lipid metabolism
Source: Gut. 2022 Jan 12;71(10):2081–92. doi: 10.1136/gutjnl-2021-325109 (PMC9484377; doi:10.1136/gutjnl-2021-325109)
Supplement: Supplementary data [file gutjnl-2021-325109supp002.pdf]

# Supplementary Materials & Methods

**"The CCT3-LINC00326 axis regulates hepatocarcinogenic lipid metabolism"**

**Søndergaard *et al.***

---

***Data and code availability***

Supplementary tables can be found here: https://figshare.com/s/2c05765158269b3b4ff2
The datasets generated in this study are available in the ArrayExpress repository, under accession numbers:
E-MTAB-8915
(reviewer link: http://www.ebi.ac.uk/arrayexpress/experiments/E-MTAB-8915, username: Reviewer_E-MTAB-8915, password: xpizwqkc),
E-MTAB-9587
(reviewer link: http://www.ebi.ac.uk/arrayexpress/experiments/E-MTAB-9587, username: Reviewer_E-MTAB-9587, password: 25ddT0yi), and
E-MTAB-9586
(reviewer link: http://www.ebi.ac.uk/arrayexpress/experiments/E-MTAB-9586, username: Reviewer_E-MTAB-9586, password: M2tY9UPH). All scripts used for bioinformatics analysis are available on Github: https://github.com/jonasns/LiveRNome.
Single molecule RNA FISH imaging files generated in this study are available here:
Treated cells: https://figshare.com/s/a83dbee52555e922ca8d
Untreated cells: https://figshare.com/s/08b0f84f2ea241b03c8d

***Identification of RBPs from prior studies***

The list of 2,282 proteins that can bind RNA in liver was generated by combining information from published datasets [1,2,11–19,3–10]. The list includes both canonical and non-canonical RBPs defined by whether a known RBD is present.

***Cell culture***

All assay plates, dishes and culture flasks were purchased from Sarstedt. HepG2 and Huh7 human HCC cell lines were obtained from American Type Culture Collection (ATCC, Rockville, MD) with certified genotype. Huh7 cells constitutively expressing tubulin-GFP [20] were kindly provided by Prof. Bartenschlager (University of Heidelberg). All cell lines were mycoplasma free when periodically tested with Mycoplasmacheck (Eurofins Genomics). To ensure authenticity, cell lines were initially genotyped by short-tandem repeat genetic profiling (STR) using the Power-Plex_16HS_Cell Line panel and analyzed using Applied Biosystems Gene Mapper ID v3.2.1 software by the external provider Genetica DNA Laboratories (LabCorp Specialty Testing Group) and continuously assessed phenotypically. Huh7 and HepG2 cells were cultured in T75 flasks at 37°C and 5% $CO_2$ atmosphere using Dulbecco's Modified Eagle Medium (DMEM) supplemented with 1/100 Penicillin/Streptomycin (P/S, Sigma) and 10% fetal bovine serum (Hyclone, GE healthcare).

Huh7 cells were maintained by splitting 1/6 three times a week, HepG2 by splitting 1/4 three times a week. This was done by aspirating the medium, gently washing the cells with phosphate buffered saline (PBS) without $Mg^{2+}$ (Sigma) and detaching them with 2mL of a trypsin-EDTA solution (Sigma) for 3-5min. Trypsin was inactivated with a minimum of 10-fold surplus of culture medium before a cell fraction was passaged. To ensure authenticity, cell lines were initially genotyped by short-tandem repeat genetic profiling (STR) using the PowerPlex_16HS_Cell Line panel and analyzed using Applied Biosystems Gene Mapper ID v3.2.1 software by the external provider Genetica DNA Laboratories (LabCorp Specialty Testing Group) and continuously assessed phenotypically.

***Patient material***

Tissue specimens were obtained under informed consent from 24 patients undergoing liver resection of HCC from the Australian Victorian Biobank, according to the approval of local ethics committee (application number: 2010/541-31/1 and 2017/719-31/2). The patients taking part in this study were 75% males and 25% females, and presented with HCC from Hepatitis B virus infection, non-alcoholic fatty liver disease, alcoholic steatohepatitis, hereditary haemochromatosis and other HCC triggering conditions. See patient information on ArrayExpress E-MTAB-8915 (reviewer link: http://www.ebi.ac.uk/arrayexpress/experiments/E-MTAB-8915, username: Reviewer_E-MTAB-8915, password: xpizwqkc). It was not appropriate or possible to involve patients or the public in the design, or conduct, or reporting, or dissemination plans of our research.

***Survival analysis of TCGA data***

Survival analysis was done in R (v. 3.4.3) using survival (v. 3.1-8) and survminer (v. 0.4.6). The script is available at: https://github.com/jonasns/LiveRNome. A total of 377 patients were used for the analysis, and divided into terciles based on normalized htseq-counts of mRNA expression. Metadata and raw htseq-counts were acquired from https://www.cancer.gov/tcga (accessed on 2017.02.17).

***siRNA-mediated KD of RBPs***

At 70-80% confluency, cells were harvested by trypsination and subjected to electroporation. Small interfering RNA (siRNA) POOLs targeting *PEG10*, *HIST1H1C*, *KPNA2*, *PKM*, *NQO1*, *TOP2A*, *CCT3*, *STMN1*, *DDX39A*, and *IGF2BP1* was used (Thermo Scientific). siRNA ON-TARGETplus Non-Targeting siRNA#1 (Thermo Scientific) was used as control. Cells were washed twice in PBS and once in OptiMEM without phenol red (Invitrogen). A total of 5µg (18.8µL of a 20µM stock) siRNA was transferred to a 4mm cuvette (Bio-Rad), and 5 million cells were added in 200µl OptiMEM and incubated for 3min before being pulsed with an exponential decay pulse at 300V, 250µF, in a Genepulser II (Bio-Rad). Immediately after electroporation, the cells were transferred to pre-heated (37°C) phenol red-free DMEM culture medium supplemented with 10% (v/v) FBS and without the addition of antibiotics. Electroporation conditions were optimized using DY547-siGLO RISC-free control siRNA (Dharmacon) and flow cytometry (FACSNavios, Beckman Coulter, Navios Cytometry List Mode Data Acquisition and Analysis Software version 1.3). Optimization results were analyzed using FlowJo (v. 8.2). Gating strategy can be found in Fig. S4.

***RNA extraction and DNase-treatment***

All reagents and devices were purchased from ThermoFisher Scientific, if not stated otherwise. Upon cell harvest, 700µL Qiazol (Qiagen) was directly added onto the cells on ice and mixed. At this point, the cell extract was either stored at -80°C or directly added 140µL chloroform. This Qiazol/chloroform mixture was shaken for 30 sec and incubated at RT for 2.5min, before centrifugation at 9,000g for 5min at 4 °C. The mixture separated into a lower chloroform-phenol phase, an interphase and an upper, aqueous phase. The latter was carefully transferred to a new reaction tube before adding 1 volume isopropanol, inverting the tube 5 times followed by 10min incubation at RT. The mixture was centrifuged at 9,000g and 4°C for 10min and the supernatant discarded. The pellet was washed carefully using 700µL cold 70% ethanol, flicking the tube and centrifuged at 15,000g and 4°C for 5min. The supernatant was discarded entirely, and the pellet air-dried for 5min, before resuspension in 20–50µL nuclease-free water (NF-$H_2O$, Ambion). RNA concentration was determined by nanodrop (Nanodrop 2000c). When possible, 10µg RNA was mixed with NF-$H_2O$, 5µL TurboDNase buffer, 1µL TurboDNase and 1µL RNase Inhibitor (RiboLock, Invitrogen) in a total volume of 50µL and incubated at 37°Cfor 30min. Afterwards, 5µL Turbo DNase Inactivation reagent was added to the tube and incubated at RT for 5min. During incubation, the tube was occasionally flicked. The tube was centrifuged at 10,000g and 4°C for 2min and the supernatant carefully transferred to a new reaction tube. In order to achieve higher purity, the RNA was precipitated overnight. This was done by adding 19µL 3M sodium acetate (pH 5.2), 1µL GlycoBlue (Ambion), 135µL NF-H2O and 600µL ice-cold 99.8% ethanol, homogenizing and storing the tube at -80°C. The next day, the sample was centrifuged at 12,000g for 30min, the supernatant discarded, and the pellet washed twice with 700µL 70% ethanol. The pellet was air-dried, resuspended in 25µL NF-H2O and the RNA concentration determined by nanodrop.

For the Australian HCC cohort, total RNA from the liver biopsies was extracted using the RNeasy mini kit (Qiagen) and treated with DNase RNase free kit (Qiagen).

***cDNA synthesis and qPCR***

All reagents and devices were purchased from ThermoFisher Scientific, if not stated otherwise. 1-2µg of pure DNase-treated RNA was added 1µL random primers (250ng/µL) and 1µL dNTP Mix (10m each) to a final volume of 13.5µL. The sample was incubated in a thermocycler (ProFlex PCR) at 65°C for 5min. Afterwards, 7µL of a mastermix containing 1µL RNase-Inhibitor, 4µL First-Strand Buffer, 1µL 0.1M DTT and 0.5µL SuperScript II reverse transcriptase were added. Samples were incubated in the thermocycler at 25°C for 10min, 42°C for 50min and 70°C for 15min and a final hold at 4°C.

The cDNA used for qPCR was diluted 1/10 to 1/25 depending on the experiment. 3.6X Master mixes were prepared, containing 3.6µL of primer mix (forward and reverse primer, 2.5µM each), 10.8µL NF-H2O and 18µL SYBR-Green Mix (PowerUp) per sample. 3.6µL of diluted cDNA was added to a 96-well PCR plate and 32.4µL of the prepared master mix added. After mixing, three times 10µL were transferred to a 384-well plate to obtain three technical replicates per sample. After sealing the plate and a short centrifugation, it was run on Real-Time-PCR machine (QuantStudio 5 or CFX384 (Bio-Rad)). The following

parameters were set: initial heating steps of 3min at 50°C followed by 3min at 95°C. 40 cycles of 15sec at 95°C and 30sec at 60°C. Melt curve: 60-95°C using 0.5°C increments every 5sec. Quantitative PCR data was checked for correct amplification and dissociation of the products. mRNA levels of the genes of interest were normalized to mRNA levels of the housekeeping genes *GAPDH* or *ACTB* and were calculated according to the ddCT method.

***Primer design***

Exon sequences of desired genes were obtained from NCBI Reference Sequence Database (RefSeq) and used as input on www.primer3.ut.ee (version 4.1.0). The following parameters were set: Tm 60-65°C with optimum of 60°C, GC-content 40-60% with optimum of 50% and product size range from 100-300 bp. Primer results were validated using the in-silico PCR tool in UCSC Genome Browser (Human, Assembly: Dec. 2013 – GRCh38/hg38, target: genome assembly). Whenever possible, the primers were designed to be intron-spanning. Primers used can be found in Table S14.

***MTT proliferation assay***

Directly after electroporation, 5,000 Huh7 or 7,000 HepG2 cells were seeded into 96 well plates in triplicates. Cells were assayed between day 1 and day 7 every 48h. For this purpose, the medium was aspirated and a mixture of 10µL MTT (Methylthiazolyldiphenyl-tetrazolium bromide, Sigma, 4mg/mL in 1X PBS) and 60µL culture medium added to the cells, before incubating at 37°Cand 5% $CO_2$ atmosphere for 1 h. After carefully removing the medium, 100µL of MTT-lysis buffer per well (for 100mL MTT lysis buffer: 90mL isopropanol, 2.5mL 10% SDS, 4mL HCl, 3.5mL H2O) was added onto the cells and incubated on an orbital shaker for 15-30min. The lysate was resuspended, the bottom of the assay plate wiped with ethanol and the absorption at 595nm measured on a plate reader (Spectramax i3x, Molecular Devices). Background values were subtracted from the obtained values before normalization to the average value of the plate in order to compare between different experiments.

***Viability measures***

Supernatants containing non-adherent cells and adherent cells (harvested by trypsination) were harvested directly into FACS tubes. For 7-AAD single staining, cells were washed 1X in PBS with 1% BSA (Sigma), followed by staining in 100µL buffer with 5µL 7-AAD Viability Staining Solution (eBioscience) on ice in the dark for 15min. Cells were acquired directly on a flow cytometer (FACSNavios, Beckman Coulter) without washing away the staining buffer. For detection of early apoptosis, cells were washed twice in PBS (without BSA), and resuspended in 100µL Annexin V binding buffer (10mM HEPES (pH 7.4), 150mM NaCl, 2.5mM $CaCl_2$ in $H_2O$) containing 5µL 7AAD and 5µL Annexin V-APC (eBioscience). Cells were stained on ice in the dark for 30min and acquired as for 7-AAD single staining. Data were acquired by flow cytometry (FACSNavios, Beckman Coulter, Navios Cytometry List Mode Data Acquisition and Analysis Software version 1.3) and analyzed using FlowJo (v. 8.2). Gating strategy can be found in Fig. S6.

***Sequencing of RNA libraries***

The library preparation was carried out using the Illumina TruSeq® Stranded RNA Library Prep Kit v2 (dual index) according to the manufacturer's instruction and ribosomal depletion was achieved with Ribo-Zero Gold. The quality of every cDNA library was determined on high-sensitivity DNA chips (Agilent Bioanalyzer) according to the manufacturer's protocol. Libraries were quantified with the KAPA-SYBR FAST qPCR kit (Roche). The sequencing run was performed either with the NextSeq 500/550 High Output v2 kit (Illumina) for 150 cycles, paired end, on a Illumina NextSeq 500 or HiSeq Rapid SBS Kit v2 for 50 cycles, single end on a Illumina HiSeq2500. All raw data (fastq files) are accessible under ArrayExpress:
E-MTAB-9587
(reviewer link: http://www.ebi.ac.uk/arrayexpress/experiments/E-MTAB-9587,
username: Reviewer_E-MTAB-9587, password: 25ddT0yi) and
E-MTAB-9586
(reviewer link: http://www.ebi.ac.uk/arrayexpress/experiments/E-MTAB-9586,
username: Reviewer_E-MTAB-9586, password: M2tY9UPH)

***RNA-seq data QC and processing***
Next generation sequencing read quality were assessed with FastQC (v. 0.11.5) and RseQC (v. 2.6.4). Adaptor sequences were trimmed and low-quality reads removed using Trimmomatic (v. 0.36). All sequencing reads aligning (HiSAT2, v. 2.1.0) to annotated ribosomal RNA genes were discarded. High-quality and ribosomal RNA depleted sequencing reads were aligned to the genome using HiSAT2. Novel RNA transcripts were identified using StringTie (v. 1.3.3). Using sorted bam files (Samtools v. 1.5), the number of aligned reads were counted (featurecount in subread package v. 1.5.2) for both annotated transcripts and novel references generated with StringTie. After normalization (TMM: trimmed mean of M-values), a differential gene expression analysis (edgeR v. 3.20.7) was performed in R (v. 3.4.3). For the two patient cohorts (TCGA and Australia), DESeq (v. 1.14.1 in bioconductor v. 3.4) was used for differential gene expression analysis. In general, we use edgeR for n<6 and DESeq for n>6 replicates. Significant DE genes was distinguished by a false discovery rate (FDR) under 0.05 or 0.01. Gene ontology analysis was performed in R (v. 3.4.3) with clusterProfiler (v. 3.6.0) and org.Hs.eg.db (v. 3.5.0), with p values corrected by the Benjamini-Hochberg FDR procedure. Additionally, the following dependent package versions were installed: DOSE (v. 3.4.0), AnnotationDbi (v. 1.40.0), IRanges (v. 2.12.0), S4Vectors (v. 0.16.0), BiocGenerics (v. 0.24.0), and Biobase (v. 2.38.0). All scripts are available on Github: https://github.com/jonasns/LiveRNome.

***CRISPRa vector generation and cell electroporation***
SP-dCas9-VPR [21] were acquired from Addgene plasmid # 63798 (deposited by George Church). Three gRNAs targeting within the 300 bp upstream region of the transcriptional start site were designed for each lincRNA using the Broad Institute design tool (https://portals.broadinstitute.org/gpp/public/analysis-tools/sgrna-design, accessed on 2018.01.08). Each gRNA was checked for off-target effects using both the Broad Institute tool's internal checkup and by BLATing them in UCSC genome browser. gRNAs were assembled into SP-dCas9-VPR using Gibson assembly (NEB). Non-targeting gRNAs were

from [22] and is not found anywhere in the human genome. The gRNA sequences can be found in Table S14. HepG2 and Huh7 cells were electroporated using the NEON electroporation system (Invitrogen) as previously described [20]. Transfection efficiency was checked by qPCR of target genes.

***Lipid experiments***

Assays for lipid peroxidation (MDA) (Abcam, ab233471), DCFDA cellular ROS detection (Abcam, ab113851) and hepatic lipid accumulation/steatosis (ORO, Abcam, ab133131) were conducted according to the manufacturers' protocols. For the MDA assay, cells were lysed directly in the culture dish with a modified 4°C cold RIPA buffer (10mM Tris-HCl pH 8.0, Sigma), 150mM NaCl (Sigma), 1mM Na2EDTA (Fisher), 1mM EGTA (VWR), 1% NP-40 (Sigma), 1% sodium deoxycholate (VWR), 0.1% SDS (Sigma). Lysates were kept on ice unless otherwise stated. The MDA assay was performed 48h after transfection, while for the ROS and ORO assays, cells were collected, counted and re-plated in equal numbers 48h after transfection, followed by readout the day after. The ROS assay was performed in 96wp, while ORO was performed in 24wp, until after dye extraction, which was transferred to 96wp. All experiments were read out on a Spectramax i3x.

***Zebrafish xenograft experiments***

Zebrafish experimentation was performed at the zebrafish core facility at Comparative Medicine, Karolinska Institute, Sweden. Husbandry and breeding were in accordance to the ethical permit Dnr 14049-2019 approved by the Stockholm North Ethical Board. Zebrafish experimentation in embryos younger than five days are excluded from the normative on animal testing by the EU directive 2010/63/EU.

Huh7 cells constitutively expressing TUBULIN-GFP [23] were cultured identical to standard Huh7 cells, except for adding 10ug/mL Blasticidin S HCl (Thermo Fisher) to the growth medium. TUBULIN-GFP Huh7 cells were grown to 80% confluency in a T75 flask. Medium was removed and cells were washed twice with pre-warmed 1xPBS. Five mL of pre-warmed 1xPBS was added to the cell culture followed by 20µL of Vybrant DiICell-labeling solution (Thermo Fisher). After incubating for 20min at 37°C excessive dye was washed away with 1xPBS and cells were harvested using Accutase (Sigma). Accutase was inactivated with 10-fold excess culture medium and filtered through a pre-washed 40µM cell strainer (Sigma). After pelleting (500g, 4min) cells were resuspended in 1mL of 2% (w/v) polyvinylpyrrolidone (Sigma) in PBS and transported on ice to the zebrafish core facility. Cells were re-pelleted, resuspended by flicking the tube, and transplanted into the perivitelline space of 48h old zebrafish embryos (AB genotype). Tumor growth was followed by daily by epifluorescence microscopy (Leica) from day 1 to 5 after injection. Tumor area was quantified using Fiji in ImageJ (v. 1.52).

***Promoter analysis***

Promoter analysis was done using the gimmeroc command in GimmeMotifs v0.13.1 with fasta sequences 400bp upstream of DEG transcription start site as input and 1000 random sequences with equal length and GC content from hg38 as background.

***RNA immunoprecipitation followed by quantitative PCR (RIP-qPCR)***

RIP-qPCR was done using the Magna RIP RBP IP kit (Sigma) according to the manufacturer's instructions. Cell lysates were prepared from Huh7 or HepG2 HCC cell lines with *LINC00326*-OE, and immunoprecipitated using CCT3 Ab 10571-1-AP (Proteintech, lot #00021192) rotating overnight at 4°C. HULC was used as a negative control.

***Subcellular fractionation***

Subcellular fractions were prepared as described in [17]. Cultured cells at an approximate confluence of 90 % were harvested, counted, washed with cold PBS and ~0.5x107 cells were transferred to a reaction tube. Cells were centrifuged at 500×g and 4°C for 5 min, the supernatant discarded, and the cells very gently resuspended in 380 µL hypotonic lysis buffer (HLB) containing 100 U RNase Inhibitor. After 10 min incubation on ice, the lysate was centrifuged at 1000×g and 4°C for 3 min. The supernatant containing the cytoplasmic fraction was carefully transferred to a new reaction tube, while leaving the pellet on ice. 1 mL RNA precipitation solution was directly added to the supernatant, and stored at -20°C for at least 1 h. The pellet containing the nuclear fraction was washed three times by addition of 1 mL HLB and centrifugation at 200×g at 4°C for 2 min. Subsequently, the pellet was dissolved in 1 mL Qiazol and kept on ice for short- or at -80°C for long-term storage. After 1 h of incubation at -20°C, the cytosolic fraction was vortexed for 30 sec and centrifuged at 18,000×g and 4°C for 15 min. After discarding the supernatant, the pellet was washed with ice-cold 70% ethanol, centrifuged at 18,000×g and 4°C for 5 min and air dried. Subsequently, the pellet was resuspended in 1 mL Qiazol and mixed until the pellet was fully dissolved. RNA from the cytosolic and nuclear fractions was extracted and the reverse transcription followed by quantitative PCR was performed.

***Single-molecule RNA FISH***

Cells were grown on coverslips in 12-well plates and were left untreated or were transfected with *LINC00326* or appropriate negative control guides with CRISPRa. The cells were briefly washed with 1xPBS (diluted in RNase-free H2O) and fixed with 75% methanol + 25% acetic acid at RT for 10 min. Following fixation, cells were washed twice with 1xPBS. The coverslips were briefly rehydrated with Wash Buffer A (SMF-WA1-60, Biosearch) (Formamide, Thermo Fischer Scientific, AM9342) before FISH. The Stellaris FISH Probes (LINC00326 exonic probes, Q570) were designed using the Stellaris online RNA FISH probe designer (Stellaris Probe Designer, version 4.2), which was produced by LGC Biosearch Technologies. The LINC00326 probes were added to the hybridization buffer (SMF-HB1-10, LGC Biosearch Technologies) at a final concentration of 250 nM, and hybridization was carried out in a humidified chamber at 37 °C overnight. The following day, the cells were washed twice with Wash Buffer A (SMF-WA1-60, LGC Biosearch Technologies) at 37 °C for 30 min. The second wash contained 4,6-diamidino-2-phenylindole for nuclear staining (5 µg/ml, Merck-Sigma, D9542). The coverslips were then briefly washed with wash buffer B (SMF-WB1-20, LGC Biosearch Technologies) for 5 min at RT, equilibrated 5 min in base glucose buffer (2x SSC, 0.4% glucose solution 49163, Merck-Sigma, in RNase-free H2O), and then incubated 5 min in Base Glucose buffer supplemented with 1:100 dilution of glucose oxidase (stock 3.7 mg/mL, G2133, Merck-Sigma) and catalase (stock 4 mg/mL,

219261-100KU, Merck-Sigma). Finally, the coverslips were mounted with ProlongGold Antifade Mountant (P36934, Thermo Fischer Scientific). Images were captured with wide-field DeltaVision microscope (Applied Precision, LLC) equipped with a Coolsnap HQ2 12 bit camera with 1x1 binning and 896x896 frame size. We acquired 20-30 optical slices depending on the thickness of the cell lines at 0.3-µm intervals using Olympus 100x (1.4 numerical aperture) oil immersion objectives. Deconvolution of three-dimensional stacks was performed with built-in DeltaVision SoftWoRx Imaging software (Applied Precision, LLC). Maximum intensity projections were generated in Fiji and subjected for manual quantification using Fiji. The sequences of LINC00326 RNA FISH probe can be found in Table S14.

**Supplementary Materials & Methods References**


1 Castello A, Fischer B, Eichelbaum K, *et al.* Insights into RNA biology from an atlas of mammalian mRNA-binding proteins. *Cell* 2012;**149**:1393–406. doi:10.1016/j.cell.2012.04.031

2 Castello A, Fischer B, Frese CK, *et al.* Comprehensive Identification of RNA-Binding domains in human cells. *Mol Cell* 2016;**63**:696–710. doi:10.1016/j.molcel.2016.06.029

3 Gerstberger S, Hafner M, Tuschl T. A census of human RNA-binding proteins. *Nat Rev Genet* 2014;**15**:829–45. doi:10.1038/nrg3813

4 Baltz AG, Munschauer M, Schwanhäusser B, *et al.* The mRNA-bound proteome and Its global occupancy profile on protein-coding transcripts. *Mol Cell* 2012;**46**:674–90. doi:10.1016/j.molcel.2012.05.021

5 Caudron-Herger M, Rusin SF, Adamo ME, *et al.* R-DeeP: proteome-wide and quantitative identification of RNA-dependent proteins by density gradient ultracentrifugation. *Mol Cell* 2019;**75**:184-199.e10. doi:10.1016/j.molcel.2019.04.018

6 Bao X, Guo X, Yin M, *et al.* Capturing the interactome of newly transcribed RNA. *Nat Methods* 2018;**15**:213–20. doi:10.1038/nmeth.4595

7 Perez-Perri JI, Rogell B, Schwarzl T, *et al.* Discovery of RNA-binding proteins and characterization of their dynamic responses by enhanced RNA interactome capture. *Nat Commun* 2018;**9**. doi:10.1038/s41467-018-06557-8

8 Mullari M, Lyon D, Jensen LJ, *et al.* Specifying RNA-Binding Regions in Proteins by Peptide Cross-Linking and Affinity Purification. *J Proteome Res* 2017;**16**:2762–72. doi:10.1021/acs.jproteome.7b00042

9 Trendel J, Schwarzl T, Horos R, *et al.* The Human RNA-Binding Proteome and Its Dynamics during Translational Arrest. *Cell* 2019;**176**:391-403.e19. doi:10.1016/j.cell.2018.11.004

10 Brannan KW, Jin W, Huelga SC, *et al.* SONAR Discovers RNA-Binding Proteins from Analysis of Large-Scale Protein-Protein Interactomes. *Mol Cell* 2016;**64**:282–93. doi:10.1016/j.molcel.2016.09.003

11 Treiber T, Treiber N, Plessmann U, *et al.* A Compendium of RNA-Binding Proteins that Regulate MicroRNA Biogenesis. *Mol Cell* 2017;**66**:270-284.e13. doi:10.1016/j.molcel.2017.03.014

12 He C, Sidoli S, Warneford-Thomson R, *et al.* High-Resolution Mapping of RNA-Binding Regions in the Nuclear Proteome of Embryonic Stem Cells. *Mol Cell* 2016;**64**:416–30. doi:10.1016/j.molcel.2016.09.034

13 Conrad T, Albrecht AS, De Melo Costa VR, *et al.* Serial interactome capture of the human cell nucleus. *Nat Commun* 2016;**7**. doi:10.1038/ncomms11212

14 Beckmann BM, Horos R, Fischer B, *et al.* The RNA-binding proteomes from yeast to man harbour conserved enigmRBPs. *Nat Commun* 2015;**6**. doi:10.1038/ncomms10127
15 Sundararaman B, Zhan L, Blue SM, *et al.* Resources for the Comprehensive Discovery of Functional RNA Elements. *Mol Cell* 2016;**61**:903–13. doi:10.1016/j.molcel.2016.02.012
16 Hendrickson D, Kelley DR, Tenen D, *et al.* Widespread RNA binding by chromatin-associated proteins. *Genome Biol* 2016;**17**. doi:10.1186/s13059-016-0878-3
17 Cook KB, Kazan H, Zuberi K, *et al.* RBPDB: A database of RNA-binding specificities. *Nucleic Acids Res* 2011;**39**. doi:10.1093/nar/gkq1069
18 Ray D, Kazan H, Cook KB, *et al.* A compendium of RNA-binding motifs for decoding gene regulation. *Nature* 2013;**499**:172–7. doi:10.1038/nature12311
19 Kwon SC, Yi H, Eichelbaum K, *et al.* The RNA-binding protein repertoire of embryonic stem cells. *Nat Struct Mol Biol* 2013;**20**:1122–30. doi:10.1038/nsmb.2638
20 Søndergaard JN, Geng K, Sommerauer C, *et al.* Successful delivery of large-size CRISPR/Cas9 vectors in hard-to-transfect human cells using small plasmids. *Commun Biol* 2020;**3**:1–6. doi:10.1038/s42003-020-1045-7
21 Chavez A, Scheiman J, Vora S, *et al.* Highly efficient Cas9-mediated transcriptional programming. *Nat Methods* 2015;**12**:326–8. doi:10.1038/nmeth.3312
22 Liu SJ, Horlbeck MA, Cho SW, *et al.* CRISPRi-based genome-scale identification of functional long noncoding RNA loci in human cells. *Science* 2017;**355**:eaah7111. doi:10.1126/science.aah7111
23 Cortese M, Goellner S, Acosta EG, *et al.* Ultrastructural Characterization of Zika Virus Replication Factories. *Cell Rep* 2017;**18**. doi:10.1016/j.celrep.2017.02.014
