# Supplementary material for: CCT3-LINC00326 axis regulates hepatocarcinogenic lipid metabolism
Source: Gut. 2022 Jan 12;71(10):2081–92. doi: 10.1136/gutjnl-2021-325109 (PMC9484377; doi:10.1136/gutjnl-2021-325109)
Supplement: Supplementary data [file gutjnl-2021-325109supp003.pdf]

Supplemental Material

"The CCT3-*LINC00326* axis regulates hepatocarcinogenic lipid metabolism"
Søndergaard *et al.*

SUPPLEMENTARY FIGURES

Figure S1. Molecular characteristics of RBPs in HCC
Figure S2. Survival analysis of the TCGA-LIHC cohort
Figure S3. Estimation of hazard ratio and HCC patient survival based on RBP gene expression
Figure S4. siRNA transfection efficiency and RBP-KD perturbation effects in human HCC cells
Figure S5. Gene Ontology (GO) and KEGG analyses of deregulated genes upon RBP-KD
Figure S6. Cellular and molecular phenotype assessment upon lincRNA-OE
Figure S7. GO and KEGG analyses of deregulated genes upon OE
Figure S8. CCT3 works in a chaperoning-independent manner to interact with *LINC00326*
Figure S9. GO and KEGG analyses of deregulated genes shared upon RBP-KD and lincRNA OE
Figure S10. *LINC00326* is highly expressed in healthy testis and diminished upon cancer progression
Figure S11. Molecular model for *LINC00326* regulating lipid metabolism-associated gene expression

SUPPLEMENTARY TABLES

Table S1. hRBP list
Table S2. TCGA LIHC DEG
Table S3. AUS counts
Table S4. AUS DEG
Table S5. RBP KD RNAseq raw counts
Table S6. RBP KD RNAseq TPM
Table S7. RBP KD RNAseq DEG
Table S8. RBP KD RNAseq GO KEGG
Table S9. novel genes info
Table S10. lncRNA OE RNAseq raw counts
Table S11. lncRNA OE RNAseq TPM
Table S12. lncRNA OE RNAseq DEG
Table S13. lncRNA OE and RBP overlap GO KEGG
Table S14. Primer list
Supplementary tables and microscopic imaging files are accessible via Figshare:
https://figshare.com/s/2c05765158269b3b4ff2
https://figshare.com/s/a83dbee52555e922ca8d
https://figshare.com/s/08b0f84f2ea241b03c8d

SUPPLEMENTARY MATERIALS & METHODS

Cell-based and molecular assays as well as xenograft experiments are described in the supplementary materials and methods.

ARRAYEXPRESS ACCESSION

Australian HCC patient cohort RNA-seq: E-MTAB-8915
RBP-KD RNA-seq: E-MTAB-9587
lncRNA-OE RNA-seq: E-MTAB-9586

CODE ACCESSIBILITY

github.com/jonasns/LiveRNome

Søndergaard JN, *et al. Gut* 2022; 71:2081–2092. doi: 10.1136/gutjnl-2021-325109
